# Comparison of the efficacy of three topical antiseptic solutions for the prevention of catheter colonization: a multicenter randomized controlled study

**DOI:** 10.1186/s13054-017-1890-z

**Published:** 2017-12-21

**Authors:** Hideto Yasuda, Masamitsu Sanui, Takayuki Abe, Nobuaki Shime, Tetsuya Komuro, Junji Hatakeyama, Shohei Matsukubo, Shinji Kawano, Hiroshi Yamamoto, Kohkichi Andoh, Ryutaro Seo, Kyo Inoue, Eiichiro Noda, Nobuyuki Saito, Satoshi Nogami, Kentaro Okamoto, Ryota Fuke, Yasuhiro Gushima, Atsuko Kobayashi, Toru Takebayashi, Alan Kawarai Lefor, for Japanese Society of Education for Physicians and Trainees in Intensive Care (JSEPTIC) Clinical Trial Group

**Affiliations:** 1 Intensive Care Unit, Department of Emergency and Critical Care Medicine, Japanese Red Cross Musashino Hospital, 1-26-1, Kyounanchou, Musashino-shi, Tokyo 180-8610 Japan; 2 Department of Intensive Care Unit, Kameda General Hospital, 929 Higashi-cho, Kamogawa City, Chiba Prefecture 296-8602 Japan; 3 Department of Preventive Medicine and Public Health, Keio University School of Medicine, 35 Shinanomachi, Shinjuku-ku, Tokyo 160-8582 Japan; 4 Department of Anesthesiology and Critical Care Medicine, Jichi Medical University Saitama Medical Center, 1-847, Amanuma-cho, Omiya-ku, Saitama-shi, Saitama, 330-8503 Japan; 5 Biostatistics Unit at Clinical and Translational Research Center, Keio University Hospital, 35 Shinanomachi, Shinjuku-ku, Tokyo 160-8582 Japan; 6 Department of Emergency and Critical Care Medicine, Hiroshima University, Kasumi 1-2-3 Minami-ku, Hiroshima, 734-8551 Japan; 7 Department of Intensive Care Medicine, Yokohama City Minato Red Cross Hospital, 3-12-1, Shin-yamashita, Naka-ku, Yokohama-Shi, Kanagawa 231-8682 Japan; 8 Department of Emergency Medicine, Emergency and Critical Care Center, Kurashiki Central Hospital, 1-1-1 Miwa, Kurashiki, Okayama, 710-8602 Japan; 9 Intensive Care Unit, Department of Anesthesiology, Jikei University School of Medicine, 3-19-18, Nishishimbashi, Minato-ku, Tokyo 105-8471 Japan; 10 Department of Anesthesia, Toyonaka Municipal Hospital, 1-14-4, Shibaraha-chou, Toyonaka-shi, Osaka, 560-8565 Japan; 11 Emergency and Critical Care Department, Division of Intensive Care, Sendai City Hospital, 1-1-1, Asutonaga-chou, Taihaku-ku, Sendai-shi, Miyagi 982-8502 Japan; 12 Department of Emergency Medicine, Kobe City Medical Center General Hospital, 2-2-1, Minatojima-minamimachi, Chuo-ku, Kobe-city, Hyogo 650-0047 Japan; 13 Department of Emergency and Critical Care Medicine, National Hospital Organization Kyoto Medical Center, 1-1 Mukaihata-cho, Fukakusa, Fushimi-ku, Kyoto-shi, Kyoto 612-0861 Japan; 14 Department of Emergency Medicine, Fukuoka City Hospital, 1-13, Yoshizuka-honmachi, Hakata-ku, Fukuoka-shi, Fukuoka, 812-0046 Japan; 15 Shock and Trauma Center, Chiba-Hokusoh Hospital, Nippon Medical School, 1715 Kamagari, Inzai, Chiba 270-1694 Japan; 16 Department of Anesthesiology, Okayama Medical Center, 1711-1, Taeki, Kita-ku, Okayama-shi, Okayama 701-1192 Japan; 17 Department of Intensive Care Medicine, Tokyo Bay Urayasu Ichikawa Medical Center, 3-4-32, Toudaijima, Urayasu-shi, Chiba 279-0001 Japan; 18 Division of Infectious Diseases and Infection Control, Tohoku Medical and Pharmaceutical University Hospital, 1-12-1, Fukumuro, Miyagino-ku, Sendai, 983-8512 Japan; 19 Department of Acute Care and General Medicine, Saiseikai Kumamoto Hospital, 5-3-1, Chikami, Minami-ku, Kumamoto-shi, Kumamoto 861-4193 Japan; 20 Department of Central Laboratory and Infection Control, Takarazuka City Hospital, 4-5-1, Kohama, Takarazuka-shi, Hyogo 665-0827 Japan; 21 Department of Surgery, Jichi Medical University, 3311-1, Yakushiji, Simono-shi, Tochigi 329-0498 Japan

**Keywords:** Chlorhexidine, Povidone-iodine, Catheter-related infections, Anti-infective agents, Local, Anti-bacterial agents, Catheters

## Abstract

**Background:**

To compare the efficacy of three antiseptic solutions [0.5%, and 1.0% alcohol/chlorhexidine gluconate (CHG), and 10% aqueous povidone-iodine (PVI)] for the prevention of intravascular catheter colonization, we conducted a randomized controlled trial in patients from 16 intensive care units in Japan.

**Methods:**

Adult patients undergoing central venous or arterial catheter insertions were randomized to have one of three antiseptic solutions applied during catheter insertion and dressing changes. The primary endpoint was the incidence of catheter colonization, and the secondary endpoint was the incidence of catheter-related bloodstream infections (CRBSI).

**Results:**

Of 1132 catheters randomized, 796 (70%) were included in the full analysis set. Catheter-tip colonization incidence was 3.7, 3.9, and 10.5 events per 1000 catheter-days in 0.5% CHG, 1% CHG, and PVI groups, respectively (*p* = 0.03). Pairwise comparisons of catheter colonization between groups showed a significantly higher catheter colonization risk in the PVI group (0.5% CHG vs. PVI: hazard ratio, HR 0.33 [95% confidence interval, CI 0.12–0.95], *p* = 0.04; 1.0% CHG vs. PVI: HR 0.35 [95% CI 0.13–0.93], *p* = 0.04). Sensitivity analyses including all patients by multiple imputations showed consistent quantitative conclusions (0.5% CHG vs. PVI: HR 0.34, *p* = 0.03; 1.0% CHG vs. PVI: HR 0.35, *p* = 0.04). No significant differences were observed in the incidence of CRBSI between groups.

**Conclusions:**

Both 0.5% and 1.0% alcohol CHG are superior to 10% aqueous PVI for the prevention of intravascular catheter colonization.

**Trial registration:**

Japanese Primary Registries Network; No.: UMIN000008725 Registered on 1 September 2012

**Electronic supplementary material:**

The online version of this article (doi:10.1186/s13054-017-1890-z) contains supplementary material, which is available to authorized users.

## Background

Catheter-related bloodstream infections (CRBSI) are serious nosocomial infections associated with high mortality, prolonged intensive care unit (ICU) stay, and increased healthcare costs [1–3]. The incidence of CRBSI reportedly decreased to 0.7 cases per 1000 catheter-days [4], probably due to more widespread use of a prevention bundle, but further reduction is a meaningful and achievable goal [1].

The current Centers for Disease Control and Prevention (CDC) guidelines for preventing intravascular catheter-related infections published in 2011 [5] recommends skin preparation using >0.5% chlorhexidine solution with alcohol before central venous catheter (CVC) or arterial catheter (AC) placement and during dressing changes. Although several studies have demonstrated that chlorhexidine gluconate (CHG) solution decreases the incidence of CRBSI associated with peripheral venous catheters, CVCs, and ACs to a greater extent than 10% aqueous povidone-iodine (PVI) [6–10], few high-quality studies have compared the efficacy of 0.5% and 1% CHG in preventing catheter colonization with that of 10% PVI [11–15]. Furthermore, 2% CHG solutions are not available in several countries, including Japan. Therefore, a study to compare 0.5% and 1% CHG with 10% PVI is essential.

To identify the ideal topical antiseptic solution for clinical use, however, CRBSI itself may not be feasible as an outcome measure due to the low incidence of CRBSI. Instead, catheter colonization is a reasonable and durable surrogate endpoint [16] when performing a meaningful study with a minimum number of participants. For this reason, this study was undertaken to compare the effectiveness of three topical antiseptic solutions—0.5% CHG, 1% CHG, and 10% PVI—for the prevention of CVC and AC colonization. We hypothesize that catheter colonization incidence is lower after skin preparation using 0.5% or 1% CHG compared with the incidence after using 10% PVI.

## Methods

### Trial design

This multicenter, open-label, parallel randomized controlled study was conducted in 16 ICUs (1 December 2012 to 31 March 2014) including four university hospitals and 12 general hospitals in Japan. Nine intensive care units were medical ICUs only and seven were combined medical-surgical ICUs. The review boards of all participating institutions approved the study protocol, and patients/close relatives provided written informed consent. This study was registered with the Japanese Clinical Research Registration System of the University Hospital Medical Information Network (UMIN) (registration number UMIN000008725).

### Patients

All consecutive CVCs and ACs placed in patients ≥18 years old after admission to the ICU for the intravenous administration of medications, hemodynamic monitoring and frequent blood sampling were eligible for inclusion. Exclusion criteria included: catheters inserted before ICU admission, catheters inserted for long-term total parenteral nutrition or chemotherapy for seven or more days, patients with a history of drug allergy to the study antiseptic solutions, patients with systemic adverse events or skin reactions at the catheter insertion site, and catheters changed using a guidewire.

### Intervention/randomization

The enrolled catheters were randomly allocated to the following three groups according to the antiseptic solution used for initial and subsequent cutaneous antisepsis: 0.5% alcohol/CHG solution with 79% ethanol (Maskin Wethanol Solution 0.5% w/v; Maruishi Pharmaceutical Company, Osaka, Japan), 1.0% alcohol/CHG solution with 79% ethanol (Hexizac AL Solution 1%; Yoshida Pharmaceutical Company, Tokyo, Japan), and 10% aqueous PVI solution (10% PVI; Isodine Solution 10%; Meiji Seika Pharma Co., Tokyo, Japan). Until the randomized catheter was removed, a subsequently placed catheter in the same patient was not included the study. The same antiseptic solution was used during dressing changes and before catheter removal. Randomization was stratified by the hospital and catheter type (CVCs or ACs) using the sealed opaque envelope method with computer-generated randomized blocks of nine which was generated at the central institution and not revealed to the participating patients/physicians/nurses. Although patients/physicians/nurses were not blinded to the antiseptic solution group, the microbiologists performing the catheter-tip cultures and data analyst were blinded to this information.

### Clinical assessment/procedure and bacteriological methods

The type of catheters and insertion sites in this study were selected at the discretion of individual physicians. At the time of conducting the study, silver antiseptic or antimicrobial-impregnated catheters were unavailable in Japan.

Before catheter insertion, the site was prepared using the allocated antiseptic solution for 30 s and allowed to dry according to standardized protocols (i.e., CHG 30s; PVI 2 min). Physicians used maximal barrier precautions (i.e., sterile gloves, gowns, masks, and large drapes covering the chest), as per CDC guidelines [5]. After catheter insertion, sterile polyurethane or gauze dressings (if an exudate was observed) were placed at the insertion site. The dressings were changed every 7 days for polyurethane dressings, every 72 h for gauze dressings, or sooner if soiled or wet. No antiseptic-containing dressings were used. Physicians/nurses inspected the insertion site at ≤24-h intervals for any evidence of infection (e.g., erythema, pain, swelling, or purulent discharge).

Catheters were removed if their use was not required or if a CRBSI was suspected [e.g., fever (temperature of ≥38.5 °C); leukocytosis (leukocyte count ≥14,000 cells/mm^3^) without any apparent cause; and/or pus, extensive erythema, or tenderness at the insertion site]. Before catheter removal, the same antiseptic solution was used at the site in an identical manner.

After catheter removal, 5 cm and 1–2 cm of the distal CVC and AC tips, respectively, were immediately sent for semi-quantitative culture analysis. The catheter tip is rolled 5–7 times across a 5% sheep blood agar plate and then aerobically cultured at 35 °C–37 °C for 24 h to 7 days [17]. All isolated bacteria were identified using standard methods. When the Maki method was unavailable, the sonication technique was used. Antimicrobial susceptibility testing was performed according to the recommendations of the Clinical and Laboratory Standards Institute.

The procedures including skin site preparation and barrier precautions during catheter insertion and removal, site preparation and dressing method during the dressing change, and the catheter culture method were standardized and explained to physicians in all ICUs before the study began. Video lectures of the standardized procedures were also distributed to the study ICUs. Definitions of study parameters (e.g., acute kidney injury and diabetes) were standardized in all ICUs before the study began.

### Outcomes

The primary outcome of this study is catheter colonization incidence per 1000 catheter-days at the time of catheter removal. Secondary outcomes are CRBSI incidence, ICU length of stay, hospital mortality, and antiseptic solution-related adverse events.

### Definition of catheter colonization and catheter-related bloodstream infection

Catheter colonization was defined as ≥15 colony-forming units (CFUs) in a semi-quantitative catheter tip culture using the roll-plate technique (i.e., by rolling the catheter segment across 5% sheep blood agar plate) after 24 h [17]. In the sonication technique, the definition of catheter colonization was at the discretion of the microbiological laboratory at each study institution. In the absence of colonization at 24 h, the culture was allowed to grow for up to 7 days. CRBSI was defined according to the CDC [18] and IDSA [19] guidelines, and the definition included three major criteria (Additional file 1: Table S1).

### Statistical analysis

In a previous study, the incidence of catheter colonization with 10% PVI and 0.5% CHG was 24.8% and 15.1%, respectively, whereas another study reported an incidence of 10.2% with 1.0% CHG [12, 13]. Based on these findings, we hypothesized that the incidence of catheter colonization will be 40% lower with CHG (0.5% or 1.0%) than with 10% PVI. We assumed that the incidence of catheter colonization in the PVI group would be 25%. The sample-size estimation showed that 245 catheters are required in each of the three arms of this study to ensure 80% power and a two-sided α error of 5%.

The data is presented as the mean with standard deviation (SD) or median with interquartile range (IQR) for continuous variables and percentages for categorical variables. One-way analysis of variance (ANOVA) and the Kruskal–Wallis test or χ^2^ test and Fisher’s exact test, as appropriate, were used for comparison. The incidence of catheter colonization and CRBSI were compared among the three groups using the log-rank test. Pairwise comparisons of the antiseptic solutions were conducted using a marginal Cox proportional hazards model with a closed testing procedure, and hazard ratios (HR) and 95% confidence intervals (CIs) were calculated. In the closed testing procedure, 0.5% CHG vs. 10% PVI was tested only if 1.0% CHG vs. 10% PVI was significant and 0.5% CHG vs. 1.0% CHG was tested if both were significant. For repeated catheter insertions in a single patient, the correlation between patient factors and catheter factors should be considered. Therefore, we applied a marginal Cox regression hazard model to compare the efficacy of the antiseptic solutions despite low proportion of repeated catheter insertions (13.4%) and a minimal intra-class correlation (r = 0.02) as shown in a previous study [5]. Subgroup analyses by catheter type (CVCs or ACs) and catheter duration (≥72 h or <72 h) were planned before commencement of the study and written in the protocol.

**Fig. 1:**
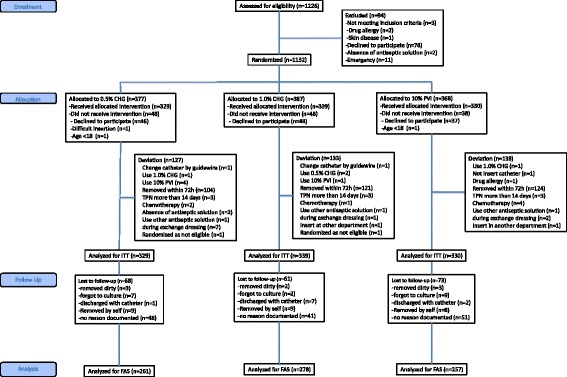
Flow chart showing randomization of skin preparation for catheter insertion. *CHG* chlorhexidine gluconate, *FAS* full analysis set, *ITT* Intention to treat, *PVI* povidone-iodine

The primary population analyzed in this study is the full analysis set (FAS), while patients without catheter colonization data were excluded from the intention-to-treat (ITT) population (Fig. 1). The groups were compared for differences in catheter site and patient characteristics missing primary outcomes, to evaluate the degree of randomization. As a sensitivity analysis to evaluate the impact of the exclusion of patients with missing data in the primary FAS analysis, multiple imputation (MI) was performed for the imputed missing data [20]. Multiple imputation (MI) is a principled method if the mechanism of missing data is missing at random. The MI by chained equations, applicable for imputations of continuous and categorical variables with a non-monotone missing pattern, was used as an imputation algorithm. The number of imputations in MI was 100, and the number of burn-ins between each imputation was 200. The incomplete response variables were catheter colonization (binary) and the duration of catheterization (continuous; log transformed). The observed covariates in the imputation were the antiseptic solution (categorical) and type of catheter (binary). In MI by chained equations, each incomplete variable was sequentially imputed, given all other variables. Analysis was performed using PROC MI and PROC MIANALYZE in SAS (SAS Inc, Cary, NC, USA). Planned interim analysis was conducted to recalculate the sample size after registration of 50 catheters in each group. The significance level for all tests was two-sided at 5%. All statistical analyses were performed using SAS, version 9.4 (SAS Inc, Cary, NC, USA).

## Results

A total of 1226 catheter placements were reviewed for eligibility, and the remaining 1132 catheters, after obtaining informed consent, were randomized to one of three solutions. Several catheters were excluded because of withdrawal of informed consent, leaving 998 (88%) catheters eligible for analysis. However, substantial numbers of catheters were not cultured for various reasons (Fig. 1). Therefore, the FAS analysis is limited to 796 (70%) catheters.

There are no significant differences in the baseline characteristics among the groups (Table 1). The characteristics of the 202 patients excluded because of the lack of catheter colonization data are similar among the three groups and between the FAS population and patients excluded from the FAS (Fig. 1, Additional file 1: Table S2 and S3). No significant differences in the insertion sites selected were observed (data not shown). The characteristics of the catheters are similar among the groups (Table 2).

**Table 1:** Characteristics of study participants

Characteristic	All N = 796	0.5% CHG N = 261	1.0% CHG N = 278	10% PVI N = 257
Age, mean (SD), years	66.2 (16.3)	65.7 (16.3)	65.9 (16.3)	67.1 (16.4)
Gender, male (n, %)	504 (63.3%)	162 (62.1%)	187 (67.3%)	155 (60.3%)
APACHE 2, mean (SD)	21.0 (8.5)	21.0 (8.6)	21.5 (8.6)	20.5 (8.4)
SAPS 2, mean (SD)	49.4 (18.6)	48.5 (19.2)	50.9 (18.2)	48.8 (18.4)
SOFA, mean (SD)	7.2 (4.0)	7.1 (3.9)	7.5 (4.3)	7.0 (3.9)
Comorbidities (n, %)				
Immunodeficiency	39 (4.9%)	10 (3.8%)	17 (6.1%)	12 (4.7%)
Steroid administration	90 (11.3%)	32 (12.3%)	35 (12.6%)	23 (9.0%)
Trauma	64 (8.0%)	27 (10.3%)	16 (5.8%)	21 (8.2%)
Cancer	84 (10.6%)	29 (11.1%)	31 (11.2%)	24 (9.3%)
Diabetes	153 (19.2%)	57 (21.8%)	51 (18.4%)	45 (17.5%)
HIV	1 (0.1%)	0 (0%)	1 (0.4%)	0 (0%)
Cirrhosis	40 (5.0%)	13 (5.0%)	14 (5.0%)	13 (5.1%)
Acute kidney injury	245 (30.8%)	85 (32.6%)	81 (29.1%)	79 (30.7%)
Chronic kidney disease	137 (17.2%)	51 (19.5%)	47 (16.9%)	39 (15.2%)
Admission category (n, %)				
Medical	645 (81.0%)	203 (77.8%)	231 (83.1%)	211 (82.1%)
Scheduled surgery	23 (2.9%)	9 (3.5%)	5 (1.8%)	9 (3.5%)
Emergency surgery	128 (16.1%)	49 (18.8%)	42 (15.1%)	37 (14.4%)
Infections before catheter insertion (n, %)	448 (56.3%)	150 (57.5%)	162 (58.3%)	136 (52.9%)
Origin of infection (n, %)				
Central nervous system	4 (0.5%)	1 (0.4%)	2 (0.7%)	1 (0.4%)
Pulmonary	195 (24.5%)	69 (26.5%)	63 (22.7%)	63 (24.5%)
Cardiovascular	5 (0.6%)	2 (0.8%)	2 (0.7%)	1 (0.4%)
Abdominal	92 (11.6%)	23 (8.9%)	44 (15.8%)	25 (9.7%)
Urinary tract	44 (5.5%)	19 (7.3%)	10 (3.6%)	15 (5.8%)
Skin	14 (1.8%)	7 (2.7%)	3 (1.1%)	4 (1.6%)
Catheter	8 (1.0%)	2 (0.8%)	5 (1.8%)	1 (0.4%)
Others	82 (10.3%)	25 (9.6%)	31 (11.2%)	26 (10.1%)
None	351 (44.2%)	112 (43.1%)	118 (42.5%)	121 (47.1%)
Antibiotics before catheter insertion (n, %)	445 (55.9%)	153 (58.6%)	155 (55.8%)	137 (53.3%)

*CHG* chlorhexidine gluconate, *PVI* povidone-iodine, *SD* standard deviation, *APACHE* acute physiology and chronic health evaluation, *SAPS* simplified acute physiology score, *SOFA* sequential organ failure assessment, *HIV* human immunodeficiency virus

**Table 2:** Characteristics of the catheters

Characteristic	All N = 796	0.5% CHG N = 261	1.0% CHG N = 278	10% PVI N = 257	*P* value
Type of catheter (n, %)					0.69
Central venous catheter	285 (35.8%)	93 (35.6%)	95 (34.2%)	97 (37.7%)	
Arterial catheter	511 (64.2%)	168 (64.4%)	183 (65.8%)	160 (62.3%)	
Insertion site (n, %)					0.10
Central venous catheter					
Internal jugular vein	265 (93.0%)	86 (92.5%)	93 (97.9%)	86 (88.7%)	
Subclavian vein	11 (3.9%)	5 (5.4%)	1 (1.1%)	5 (5.2%)	
Femoral vein	9 (3.2%)	2 (2.2%)	1 (1.1%)	6 (6.2%)	
Arterial catheter					0.20
Radial artery	481 (96.0%)	160 (97.0%)	173 (96.1%)	148 (94.9%)	
Femoral artery	15 (3.0%)	5 (3.0%)	6 (3.3%)	4 (2.6%)	
Dorsalis artery	5 (1.0%)	0	1 (0.6%)	4 (2.6%)	
Duration of catheterization, median (IQR), days	3.8 (2.0–6.7)	3.8 (2.1–6.7)	4.0 (2.0–7.0)	3.7 (2.0–6.0)	0.43
Methods for catheter culture (n, %)					0.49
Maki methods	663 (83.3%)	223 (85.4%)	227 (81.7%)	213 (82.9%)	
Sonication methods	133 (16.7%)	38 (14.6%)	51 (18.4%)	44 (17.1%)	
Dressing (n, %)					0.41
Film	668 (83.9%)	225 (86.2%)	228 (82.0%)	215 (83.7%)	
Gauze	128 (16.1%)	36 (13.8%)	50 (18.0%)	42 (16.3%)	

*CHG* chlorhexidine gluconate, *PVI* povidone-iodine, *IQR* interquartile range

**Fig. 2:**
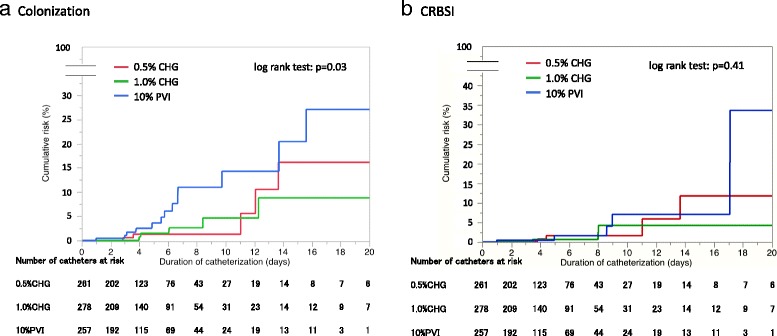
Cumulative catheter colonization and catheter-related bloodstream infection risk (Kaplan–Meier curves). **a** catheter colonization, **b** catheter-related bloodstream infection. *CHG* chlorhexidine gluconate, *PVI* povidone-iodine

The incidence of catheter-tip colonization (per 1000 catheter-days) is 3.7, 3.9, and 10.5 events in the 0.5% CHG, 1% CHG and 10% PVI groups, respectively (Table 3 and Fig. 2 (Kaplan–Meier curve) (*p* = 0.03)). Pairwise comparison of catheter colonization between groups shows that the risk is significantly higher in the 10% PVI group (0.5% CHG vs. 10% PVI: HR 0.33, 95% CI 0.12–0.95, *p* = 0.04; 1.0% CHG vs. 10% PVI: HR0.35, 95% CI 0.13–0.93, *p* = 0.04). However, there are no differences in colonization risk between the 0.5% and 1.0% CHG groups (HR 0.95, 95% CI 0.29–3.13, *p* = 0.94). Of all analyzed catheters, 134 (16.8%) were further randomized to the three solutions for second or later insertions, although the initially placed catheters were already included in the analysis. A sensitivity analysis for initially inserted catheters, excluding these 134 catheters, showed no differences in patient characteristics between the three groups, and the same conclusion was obtained, showing superiority of 0.5%/1.0% CHG over 10% PVI, as confirmed in the FAS analysis (Additional file 1: Table S4).

**Table 3:** Catheter outcomes: colonization and CRBSI

(a) No. of catheters and incidence per 1000 catheter-days					
	All N = 796	0.5% CHG N = 261	1.0% CHG N = 278	10% PVI N = 257	*P* value
Colonization					
Number of catheters (%)	24 (3.0%)	5 (1.9%)	6 (2.2%)	13 (5.1%)	0.07
Incidence per 1000 catheter-days, n (95% CI)	5.8 (3.5–8.2)	3.7 (0.5–6.9)	3.9 (0.8–7.0)	10.5 (4.8–16.3)	0.03
CRBSI					
Number of catheters (%)	13 (1.6%)	4 (1.5%)	3 (1.1%)	6 (2.3%)	0.51
Incidence, per 1000 catheter-days, n (95% CI)	3.2 (1.4–4.9)	3.0 (0.1–5.8)	2.0 (0–4.2)	4.9 (1.0–8.8)	0.41
(b) Hazard ratio for each combination of antiseptic solution					
	Colonization		CRBSI		
	HR (95% CI)	*P* value	HR (95% CI)	*P* value	
0.5% CHG vs. 10% PVI	0.33 (0.12–0.95)	0.04	0.63 (0.18–2.22)	0.47	
1.0% CHG vs. 10% PVI	0.35 (0.13–0.93)	0.04	0.41 (0.10–1.63)	0.20	
0.5% CHG vs. 1.0% CHG	0.95 (0.29–3.13)	0.94	1.54 (0.35–6.90)	0.57	

*CHG* chlorhexidine gluconate, *PVI* povidone-iodine, *HR* hazard ratio, *CI* confidence interval, *CRBSI* catheter-related bloodstream infection

In another sensitivity analysis conducted with MIs for evaluation of the impact of missing data, the marginal Cox proportional hazards model yielded results similar to those from the FAS analysis (Additional file 1: Table S5).

Subgroup analyses by catheter type (CVCs or ACs) and duration of catheter placement (≥72 h or <72 h) show that catheter colonization incidence does not significantly differ among the groups, regardless of catheter type. However, catheter duration ≥72 h poses a greater colonization risk in the 10% PVI group than that in the 0.5% CHG group (Additional file 1: Table S6 and Additional files 2, 3, 4, 5, 6 and 7: Figure S1A-F).

Since the rates of catheter insertion in the femoral vein differed among the three antiseptic solutions used, an additional analysis was conducted excluding catheters placed in the femoral vein. The results were similar to those of the primary outcome in this study, which showed that the rate of catheter colonization increased in the 10% PVI group compared to the 0.5% and 1% CHG groups (0.5% CHG vs. 10% PVI: HR 0.35, 95% CI 0.11–0.95, *p* = 0.04; 1.0% CHG vs. 10% PVI: HR0.37, 95% CI 0.13–0.97, *p* = 0.04) (Additional file 1: Table S7).

There are no significant differences in the probability of developing CRBSI among the groups (3.0 vs. 2.0 vs. 4.9 per 1000 catheter-days in 0.5% CHG, 1.0% CHG, and 10% PVI, *p* = 0.41) (Table 3). Figure 2 shows the related Kaplan–Meier plots. Pairwise between-group comparisons of CRBSI showed no significant differences, but the risk exhibits a decreasing trend in the 0.5% and 1.0% CHG groups (0.5% CHG vs. 10% PVI: HR 0.63, 95% CI 0.18–2.22, *p* = 0.47; 1.0% CHG vs. 10% PVI: HR 0.41, 95% CI 0.10–1.63, *p* = 0.20). Similar results were obtained in other subgroup analyses (Additional file 1: Table S6). A summary of patient characteristics and outcomes in patients with/without colonization or CRBSI and antibiotics before catheter insertion are shown in Additional file 1: Table S8 and S9.

Microorganisms isolated from each colonized catheter and blood in patients diagnosed with CRBSI are shown in Additional file 1: Table S10 and S11. There are no significant differences in microorganism type that caused catheter colonization and CRBSI between the groups. Systemic and local unknown serious adverse events were not observed in any of the three groups (Additional file 1: Table S12).

## Discussion

The results of this open-label, randomized controlled study show that alcohol/0.5% CHG and 1.0% CHG were superior to aqueous 10% PVI for the prevention of CVC and AC colonization, but the incidence of CRBSI is similar among the groups. The use of 10% PVI with catheters in situ for ≥72 h increases the risk of catheter colonization, whereas there was no difference in catheter colonization rates among the three solutions tested for catheters in place <72 h.

The superiority of alcohol/1% CHG over 10% aqueous PVI or alcohol/0.5% CHG for the prevention of CRBSI has not been fully examined [11–14, 21, 22]. Also, no comparative studies have examined the effect of differences in CHG concentration. Two studies [13, 14] showed that alcohol/1.0% CHG is superior to 10% aqueous PVI, but the studies had significant methodological limitations. One [13] study was prepared as an abstract for a meeting in 2001 with no subsequent publication as a full paper. The other [14] study included patients with hematological disorders alone. The only study comparing CHG at different concentrations was conducted by Valles et al [12], reporting that both alcohol with 0.5% and aqueous 2% CHG were associated with a significantly lower incidence of catheter colonization than 10% PVI. A meta-analysis published in 2016 [23] evaluated the effects of skin antisepsis as part of CVC care for reducing CRBSIs, catheter colonization, and patient mortality and morbidity. The results of subgroup comparisons based on the solution used, concluded that chlorhexidine solution may reduce rates of CRBSI and catheter colonization compared with povidone iodine. However, this study included several types of CHG solutions, including various concentrations (0.25–2.0%) of chlorhexidine and diluents (alcohol or aqueous). To the best of our knowledge, this is the first randomized control trial to demonstrate the efficacy of alcohol/1.0% CHG and 0.5% CHG over 10% aqueous PVI in preventing catheter colonization. The results also suggest non-inferiority of 0.5% CHG to 1.0% CHG.

The results of the present study have several important implications for clinical practice. This result may be beneficial in several countries, including Japan, where 2% CHG is not approved for cutaneous application for the prevention of CRBSI. In addition, it is beneficial for patients in countries that currently use 2% CHG because the risk of adverse events (e.g., anaphylaxis) may increase with CHG concentration [24].

This study has several limitations. First, the primary outcome evaluated is catheter colonization, which is only a surrogate outcome for CRBSI. The incidence of CRBSI has decreased with time [4]. Based on this, the sample size required to demonstrate differences in CRBSI incidence is enormous, making it difficult to conduct the study in a manner that will permit verification of the effectiveness of various antiseptic solutions. According to a sample size calculation based on the incidence of CRBSI in the current study, 8460 catheters would be required (80% power and a two-sided α error of 5%). However, it has been reported that there is a strong correlation between CRBSI and catheter colonization (r = 0.8) [16]. In fact, many previous studies have not defined CRBSI as the primary outcome [11, 12, 21]. To our knowledge, the only study designating CRBSI as a primary outcome is the CLEAN study [25] published in 2016, in which a total of 5159 catheters were included. Taking feasibility into consideration, we used catheter colonization incidence as the primary outcome.

Second, this study was unable to completely fulfill the ITT analysis because some of the catheter colonization data was missing after randomization due to self-removal or contamination of the catheters (Fig. 1). This may have led to confounding by unknown factors and need of the competing risk analysis. However, efforts were made to minimize this by confirming that the demographics of the excluded patients did not differ between the three groups and between the FAS and the excluded patients (Additional file 1: Table S2 and S3). We also conducted between-group comparison, with multiple imputation of missing data to perform the ITT analysis. The results using a marginal Cox proportional hazards model were similar to those obtained using FAS analysis. In the current study, the multiple imputation analysis is considered equivalent to the competing risk analysis to avoid overestimation or underestimation of the true effects. All these analyses support the robustness of our results.

Third, patient and physician blinding was not possible because the antiseptic solutions have different colors. However, a detailed study protocol, including handling of antiseptic solutions, skin preparation, and catheter insertion technique was pre-defined, and distributed to each institution. The data manager at the central hospital site was in regular communication with each hospital investigator to ensure compliance with the study protocol. In addition, the microbiologists involved in the study were blinded to the solution used. Therefore, we believe that the lack of blinding did not significantly affect the study results.

Fourth, in this study, randomization was performed at the catheter level and not at the patient level, and this may have influenced the analysis of repeated measurements. Since 16.8% of the catheters in this study represent repeat insertions in several patients, the primary outcome may have been affected because of intra-class correlations. However, taking a previous large randomized controlled study [25] (in which repeated measurements were conducted and the intra-class correlation was 0.02) into consideration, we believe that the repeated measurements in this study had little impact on the results because we were analyzing using a marginal Cox regression model. We conducted a sensitivity analysis including only the first catheter inserted in each patient, and no differences in patient characteristics were observed among the three groups. The results are similar to that those obtained using the FAS analysis.

Finally, the effectiveness of alcohol/CHG and PVI-alcohol were not compared because PVI-alcohol has not been approved for use in skin disinfection of the catheter insertion site in Japan. It has previously been reported that alcohol/2% CHG provides greater protection against short-term catheter-related infections than PVI-alcohol [25]. Further studies are necessary to verify differences in the efficacy of alcohol/0.5% or 1.0% CHG and 5% PVI-alcohol for the prevention of CRBSI.

## Conclusions

The results of this trial demonstrate that both alcohol/0.5% and 1.0% CHG are superior to 10% aqueous PVI for the prevention of catheter colonization. Hospitals where higher chlorhexidine concentrations are not permitted can use alcohol/0.5% CHG as an alternative to alcohol/1% CHG for the prevention of CRBSI. However, the optimal chlorhexidine concentration for the prevention of CRBSI has not yet been elucidated.

## Additional files

Additional file 1: Table S1. Definition of catheter-related bloodstream infection. **Table S2.** Summary of patient characteristics and outcomes in patients excluded from the FAS in the three study groups. **Table S3.** Comparison of patient characteristics of the FAS population and patients excluded from the FAS. **Table S4.** Catheter outcomes for the first catheter inserted in each patient (repeat insertions in 16.8% of patients). **Table S5.** Hazard ratio for each combination of antiseptic solution after multiple imputations. **Table S6.** Subgroup analysis for colonization and catheter-related blood stream infections. **Table S7.** Catheter outcomes for central venous catheters excluding those inserted in the femoral vein (sensitivity analysis). **Table S8.** Summary of patient characteristics and outcomes in patients with/without colonization and antibiotics before catheter insertion. **Table S9.** Summary of patient characteristics and outcomes in patients with/without catheter-related blood stream infection and antibiotics before catheter insertion. **Table S10.** Microorganisms isolated from catheter-tips in the three study groups. **Table S11.** Microorganisms isolated in catheter-related bloodstream infections in the three study groups. **Table S12.** Adverse events. **Table S13.** Patient outcomes. **Table S14.** Proportion of missing data for each variable in the three study groups. **Table S15.** The specific names of all ethical bodies that approved this study in various participating sites. (DOCX 77 kb)

Additional file 2: Figure S1. **(A)** Colonization of central venous catheters. Cumulative catheter colonization and catheter-related bloodstream infection risk in each subgroup (Kaplan–Meier curves). *CHG* chlorhexidine gluconate, *PVI* povidone-iodine. (JPG 108 kb)

Additional file 3 Figure S1. **(B)** Colonization of arterial catheters. Cumulative catheter colonization and catheter-related bloodstream infection risk in each subgroup (Kaplan–Meier curves). *CHG* chlorhexidine gluconate, *PVI* povidone-iodine. (JPG 102 kb)

Additional file 4 Figure S1. **(C)** Catheter-related bloodstream infection of central venous catheters. Cumulative catheter colonization and catheter-related bloodstream infection risk in each subgroup (Kaplan–Meier curves). *CHG* chlorhexidine gluconate, *PVI* povidone-iodine. (JPG 116 kb)

Additional file 5: Figure S1. **(D)** Catheter-related bloodstream infection of arterial catheters. Cumulative catheter colonization and catheter-related bloodstream infection risk in each subgroup (Kaplan–Meier curves). *CHG* chlorhexidine gluconate, *PVI* povidone-iodine (JPG 108 kb)

Additional file 6: Figure S1. **(E)** Catheter colonization after 72 h. Cumulative catheter colonization and catheter-related bloodstream infection risk in each subgroup (Kaplan–Meier curves). *CHG* chlorhexidine gluconate, *PVI* povidone-iodine. (JPG 107 kb)

Additional file 7: Figure S1. **(F)** Catheter-related bloodstream infections after 72 h. Cumulative catheter colonization and catheter-related bloodstream infection risk in each subgroup (Kaplan–Meier curves). *CHG* chlorhexidine gluconate, *PVI* povidone-iodine. (JPG 115 kb)

## Abbreviations

AC: Arterial catheter
ANOVA: Analysis of variance
CDC: Centers for Disease Control and Prevention
CFU: Colony-forming unit
CHG: Chlorhexidine gluconate
CI: Confidence interval
CRBSI: Catheter-related bloodstream infection
CVC: Central venous catheter
FAS: Full analysis set
HR: Hazard ratio
ICU: Intensive care unit
IQR: Interquartile range
ITT: Intention-to-treat
MI: Multiple imputation
PVI: Povidone-iodine
SD: Standard deviation
UMIN: University Hospital Medical Information Network
