# Supplementary material for: Comparison of the efficacy of three topical antiseptic solutions for the prevention of catheter colonization: a multicenter randomized controlled study
Source: Crit Care. 2017 Dec 21;21:320. doi: 10.1186/s13054-017-1890-z (PMC5740719; doi:10.1186/s13054-017-1890-z)
Supplement: Supplementary file 1 — Definition of catheter-related bloodstream infection. Table S2. Summary of patient characteristics and outcomes in patients excluded from the FAS in the three study groups. Table S3. Comparison of patient characteristics of the FAS population and patients excluded from the FAS. Table S4. Catheter outcomes for the first catheter inserted in each patient (repeat insertions in 16.8% of patients). Table S5. Hazard ratio for each combination of antiseptic solution after multiple imputations. Table S6. Subgroup analysis for colonization and catheter-related blood stream infections. Table S7. Catheter outcomes for central venous catheters excluding those inserted in the femoral vein (sensitivity analysis). Table S8. Summary of patient characteristics and outcomes in patients with/without colonization and antibiotics before catheter insertion. Table S9. Summary of patient characteristics and outcomes in patients with/without catheter-related blood stream infection and antibiotics before catheter insertion. Table S10. Microorganisms isolated from catheter-tips in the three study groups. Table S11. Microorganisms isolated in catheter-related bloodstream infections in the three study groups. Table S12. Adverse events. Table S13. Patient outcomes. Table S14. Proportion of missing data for each variable in the three study groups. Table S15. The specific names of all ethical bodies that approved this study in various participating sites. (DOCX 77 kb) [file 13054_2017_1890_MOESM1_ESM.docx]

**Additional file 1:**

**Table S1 Definition of Catheter-related blood stream infection**

| Criteria 1 | The patient had a recognized pathogen cultured from ≥1 blood cultures (the term “recognized pathogen” does not include organisms considered as common skin contaminants, i.e., those that can be cultured from ≥2 blood cultures drawn on separate occasions). |
| --- | --- |
| Criteria 2 | The patient has one or more of the following signs/symptoms: fever ≥38.0°C, chills, or hypotension, unrelated to infection at any other site. |
| Criteria 3 | The same organism is obtained from ≥1 percutaneous blood culture as well as a catheter-tip culture. |

**Table S2 Summary of patient characteristics and outcomes in patients excluded from the FAS in the three study groups**

|  | All  N = 202 | 0.5% CHG  N = 68 | 1.0% CHG  N = 61 | 10% PVI  N = 73 | P value |
| --- | --- | --- | --- | --- | --- |
| Age, mean (SD), years | 66.4 (14.8) | 65.8 (14.3) | 67.7 (15.6%) | 65.8 (14.7) | 0.99 |
| Gender, male (no., %) | 131 (64.9%) | 42 (61.8%) | 41 (67.2%) | 48 (65.8%) | 0.80 |
| APACHE 2, mean (SD) | 22.1 (9.5) | 22.2 (9.1) | 23.1 (10.0) | 21.1 (9.3) | 0.50 |
| SAPS 2, mean (SD) | 51.0 (20.3) | 51.6 (18.4) | 52.9 (21.7) | 48.7 (20.8) | 0.40 |
| SOFA, mean (SD) | 8.1 (4.3) | 8.7 (4.7) | 8.1 (4.1) | 7.6 (4.2) | 0.15 |
| Comorbidities (no., %) |  |  |  |  |  |
| Immunodeficiency | 24 (11.9%) | 8 (11.8%) | 6 (9.8%) | 10 (13.7%) | 0.79 |
| Corticosteroids administered | 33 (16.3%) | 11 (16.2%) | 9 (14.8) | 13 (17.8%) | 0.89 |
| Trauma | 13 (6.4%) | 7 (10.3%) | 2 (3.3%) | 4 (5.5%) | 0.25 |
| Cancer | 31 (15.4%) | 5 (7.4%) | 17 (27.9%) | 9 (12.3%) | 0.004 |
| Diabetes | 27 (13.4%) | 8 (11.8%) | 8 (13.1%) | 11 (15.1%) | 0.85 |
| HIV | 1 (0.5%) | 1 (1.5%) | 0 (0%) | 0 (0%) | 0.37 |
| Cirrhosis | 5 (2.5%) | 1 (1.5%) | 3 (4.9%) | 1 (1.4%) | 0.34 |
| Acute kidney injury | 53 (26.2%) | 24 (35.3%) | 12 (19.7%) | 17 (23.3%) | 0.10 |
| Chronic kidney disease | 32 (15.8%) | 12 (17.7%) | 12 (19.7%) | 8 (11.0%) | 0.34 |
| Admission category (no., %) |  |  |  |  | 0.17 |
| Medical | 161 (79.7%) | 50 (73.6%) | 55 (90.2%) | 56 (76.7%) |  |
| Scheduled surgery | 8 (4.0%) | 3 (4.4%) | 1 (1.6%) | 4 (5.5%) |  |
| Emergency surgery | 33 (16.3%) | 15 (22.1%) | 5 (8.2%) | 13 (17.8%) |  |
| Infections before catheter insertion (no., %) | 123 (60.9%) | 42 (61.8%) | 39 (63.9%) | 42 (57.5%) | 0.74 |
| Antibiotics before catheter insertion (no., %) | 118 (58.4%) | 35 (51.5%) | 40 (65.6%) | 43 (58.9%) | 0.27 |
| Type of catheter (no., %) |  |  |  |  | 0.35 |
| Central venous catheter | 56 (27.7%) | 20 (29.4%) | 20 (32.8%) | 16 (21.9%) |  |
| Arterial catheter | 146 (72.3%) | 48 (70.6%) | 41 (67.2%) | 57 (78.1%) |  |
| Duration of catheterization, median (IQR), days | 2.9 (1.4–6.5) | 3.9 (1.2–8.6) | 2.9 (1.2–7.8) | 2.8 (1.4–5.6) | 0.83 |
| ICU length of stay, median (IQR), days | 5.6 (2.6–17.4) | 8.0 (3.6–30.5) | 4.4 (2.2–13.5) | 4.9 (2.4–15.4) | 0.02 |
| Hospital length of stay, median (IQR), days | 32.0 (12.6–72.5) | 38.0 (15.1–84.9) | 29.6 (12.6–45.8) | 29.0 (11.4–72.9) | 0.32 |
| ICU mortality (no., %) | 69 (34.2%) | 25 (36.8%) | 20 (32.8%) | 24 (32.9%) | 0.86 |
| Hospital mortality (no., %) | 99 (49.0%) | 34 (50%) | 30 (49.2%) | 35 (48.0%) | 0.97 |

FAS: full analysis set, CHG: chlorhexidine gluconate, PVI: povidone-iodine, HR: hazard ratio, SD: standard deviation, APACHE: acute physiology and chronic health evaluation, SAPS: simplified acute physiology score, SOFA: sequential organ failure assessment, IQR: interquartile range, ICU: Intensive Care Unit, HIV: human immunodeficiency virus

**Table S3 Comparison of patient characteristics of the FAS population and patients excluded from the FAS**

| Characteristic | Full analysis set  N = 796 | Missing data  N = 202 | P value |
| --- | --- | --- | --- |
| Age, mean (SD), years | 66.2 (16.3) | 66.4 (14.8) | 0.90 |
| Gender, male (no., %) | 504 (63.3%) | 131 (64.9%) | 0.69 |
| APACHE 2, mean (SD) | 21.0 (8.5) | 22.1 (9.5) | 0.13 |
| SAPS 2, mean (SD) | 49.4 (18.6) | 51.0 (20.3) | 0.31 |
| SOFA, mean (SD) | 7.2 (4.0) | 8.1 (4.3) | 0.006 |
| Comorbidities (no., %) |  |  |  |
| Immunodeficiency | 39 (4.9%) | 24 (11.9%) | <0.001 |
| Corticosteroids administered | 90 (11.3%) | 33 (16.3%) | 0.052 |
| Trauma | 64 (8.0%) | 13 (6.4%) | 0.45 |
| Cancer | 84 (10.6%) | 31 (15.4%) | 0.06 |
| Diabetes | 153 (19.2%) | 27 (13.4%) | 0.053 |
| HIV | 1 (0.1%) | 1 (0.5%) | 0.31 |
| Cirrhosis | 40 (5.0%) | 5 (2.5%) | 0.12 |
| Acute kidney injury | 245 (30.8%) | 53 (26.2%) | 0.21 |
| Chronic kidney disease | 137 (17.2%) | 32 (15.8%) | 0.64 |
| Admission category (no., %) |  |  | 0.73 |
| Medical | 645 (81.0%) | 161 (79.7%) |  |
| Scheduled surgery | 23 (2.9%) | 8 (4.0%) |  |
| Emergency surgery | 128 (16.1%) | 33 (16.3%) |  |
| Infections before catheter insertion (no., %) | 448 (56.3%) | 123 (60.9%) | 0.24 |
| Antibiotics before catheter insertion (no., %) | 445 (55.9%) | 118 (58.4%) | 0.52 |
| Type of catheter (no., %) |  |  | 0.03 |
| Central venous catheter | 285 (35.8%) | 56 (27.7%) |  |
| Arterial catheter | 511 (64.2%) | 146 (72.3%) |  |
| Duration of catheterization, median (IQR), days | 3.8 (2.0–6.7) | 2.9 (1.4–6.5) | 0.0505 |
| Intensive Care Unit length of stay, median (IQR), days | 7.1 (3.3–17.3) | 5.6 (2.6–17.4) | 0.10 |
| Hospital length of stay, median (IQR), days | 34.0 (15.3–70.0) | 32.0 (12.6–72.5) | 0.20 |
| Intensive Care Unit mortality (no., %) | 158 (19.9%) | 69 (34.2%) | <0.0001 |
| Hospital mortality (no., %) | 237 (29.8%) | 99 (49.0%) | <0.0001 |

CHG: chlorhexidine gluconate, PVI: povidone-iodine, SD: standard deviation, APACHE: acute physiology and chronic health evaluation, SAPS: simplified acute physiology score, SOFA: sequential organ failure assessment, IQR: interquartile range, HIV: human immunodeficiency virus

**Table S4 Catheter outcomes for the first catheter inserted in each patient (repeat insertions in 16.8% of patients)**

1. Number of catheters and incidence per 1000 catheter-days

|  | All  N = 662 | 0.5% CHG  N = 219 | 1.0% CHG  N = 222 | 10% PVI  N = 221 | P value |
| --- | --- | --- | --- | --- | --- |
| Colonization |  |  |  |  |  |
| Number of catheters (%) | 22 (3.3%) | 4 (1.8%) | 6 (2.7%) | 12 (5.4%) | 0.09 |
| Incidence, per 1000 catheter-days, n (95% CI) | 6.2 (3.6–8.8) | 3.5 (0.1–6.8) | 4.4 (09–7.9) | 11.6 (5.0–18.1) | 0.03 |
|  |  |  |  |  |  |
| CRBSI |  |  |  |  |  |
| Number of catheters (%) | 5 (0.8%) | 2 (0.9%) | 1 (0.5%) | 2 (0.9%) | 0.81 |
| Incidence, per 1000 catheter-days, n (95% CI) | 1.4 (0.2–2.6) | 1.7 (0–4.1) | 0.7 (0–2.2) | 1.9 (0–4.6) | 0.71 |
| 1. Hazard ratio for each two antiseptic solution |  |  |  |  |  |
|  | Colonization | | CRBSI | |  |
|  | HR (95% CI) | P value | HR (95% CI) | P value |  |
| 0.5% CHG vs. 10% PVI | 0.28 (0.09–0.89) | 0.03 | 0.92 (0.13–6.56) | 0.94 |  |
| 1.0% CHG vs. 10% PVI | 0.39 (0.14–1.06) | 0.06 | 0.39 (0.04–4.33) | 0.44 |  |
| 0.5% CHG vs. 1.0% CHG | 0.72 (0.20–2.56) | 0.62 | 2.36 (0.21–26.0) | 0.48 |  |

CHG: chlorhexidine gluconate, PVI: povidone-iodine, HR: hazard ratio, CI: confidence interval, CRBSI: catheter-related blood stream infection

**Table S5 Hazard ratio for each combination of antiseptic solution after multiple imputations**

| Model 1* |  | |
| --- | --- | --- |
|  | Colonization | |
|  | HR (95% CI) | P value |
| 0.5% CHG vs. 10% PVI | 0.34 (0.12–0.96) | 0.04 |
| 1.0% CHG vs. 10% PVI | 0.40 (0.14–1.09) | 0.07 |
| Model 2** |  | |
|  | Colonization | |
|  | HR (95% CI) | P value |
| 0.5% CHG vs. 10% PVI | 0.32 (0.11–0.89) | 0.03 |
| 1.0% CHG vs. 10% PVI | 0.35 (0.13–0.94) | 0.04 |

*Model 1: The incomplete response variables were catheter colonization (dichotomous) and the duration of catheterization (continuous; log transformed). The observed covariates were the antiseptic solution (dichotomous) and the type of catheter (dichotomous).

*Model 2: The incomplete response variables were catheter colonization (dichotomous) and the duration of catheterization (continuous; log transformed). The observed covariate was the antiseptic solution (dichotomous).

**Table S6 Sub-group analysis for colonization and catheter-related blood stream infections**

1. Types of catheters
2. Number of catheters and incidence per 1000 catheter-days

|  | All  N = 796 | 0.5% CHG  N = 261 | 1.0% CHG  N = 278 | 10% PVI  N = 257 | P value |
| --- | --- | --- | --- | --- | --- |
| Colonization |  |  |  |  |  |
| Central venous catheter | 285 (35.8%) | 93 (35.6%) | 95 (34.2%) | 97 (37.7%) |  |
| Number of catheters colonized | 19 (6.7%) | 5 (5.4%) | 3 (3.2%) | 11 (11.3%) | 0.06 |
| Incidence, per 1000 catheter-days, n (95% CI) | 10.7 (5.9–15.6) | 8.8 (1.1–16.4) | 4.4 (0–9.4) | 21.2 (8.7–33.8) | 0.04 |
| Arterial catheter | 511 (64.2%) | 168 (64.4%) | 183 (65.8%) | 160 (62.3%) |  |
| Number of catheters colonized | 5 (1.0%) | 0 (0%) | 3 (1.6%) | 2 (1.3%) | 0.27 |
| Incidence, per 1000 catheter-days, n (95% CI) | 2.1 (0.3–4.0) | - | 3.5 (0–7.5) | 2.8 (0–6.7) | 0.27 |
| CRBSI |  |  |  |  |  |
| Central venous catheter | 285 (35.8%) | 93 (35.6%) | 95 (34.2%) | 97 (37.7%) |  |
| Number of CRBSI | 8 (2.8%) | 4 (4.3%) | 1 (1.1%) | 3 (3.1%) | 0.39 |
| Incidence, per 1000 catheter-days, n (95% CI) | 4.5 (1.4–7.7) | 7.0 (0.1–13.9) | 1.5 (0–4.3) | 5.8 (0–12.3) | 0.34 |
| Arterial catheter | 511 (64.2%) | 168 (64.4%) | 183 (65.8%) | 160 (62.3%) |  |
| Number of CRBSI | 5 (1.0%) | 0 (0%) | 2 (1.1%) | 3 (1.9%) | 0.22 |
| Incidence, per 1000 catheter-days, n (95% CI) | 2.1 (0.3–4.0) | - | 2.4 (0–5.5) | 4.2 (0–8.9) | 0.21 |
| 1. Hazard ratio for each two antiseptic solution | |  |  |  |  |
| Colonization | Central venous catheter | | Arterial catheter | |  |
|  | HR (95% CI) | P value | HR (95% CI) | P value |  |
| 0.5% CHG vs. 10% PVI | 0.45 (0.15–1.34) | 0.15 | 0 (0-) | 1.0 |  |
| 1.0% CHG vs. 10% PVI | 0.22 (0.06–0.80) | 0.02 | 1.15 (0.19–6.92) | 0.88 |  |
| 0.5% CHG vs. 1.0% CHG | 2.04 (0.49–8.57) | 0.33 | 0 (0) | 1.0 |  |

Data were no. (%) or n (95% CI)

CHG: chlorhexidine gluconate, PVI: povidone-iodine, HR: hazard ratio, CI: confidence interval, CRBSI: catheter-related blood stream infection

1. Duration of catheterization ≥ 72 h
2. Number of catheters and incidence per 1000 catheter-days

|  | All  N = 522 | 0.5% CHG  N = 176 | 1.0% CHG  N = 182 | 10% PVI  N = 164 | P value |
| --- | --- | --- | --- | --- | --- |
| Duration of catheterization, median (IQR), days | 5.7 (3.9–8.1) | 5.4 (3.8–8.0) | 6.0 (4.0–8.6) | 5.3 (3.8–8.3) | 0.16 |
| Catheter colonization |  |  |  |  |  |
| Total, number |  |  |  |  |  |
| Number of catheters (%) | 22 (4.2%) | 4 (2.3%) | 6 (3.3%) | 12 (7.3%) | 0.052 |
| Incidence, per 1000 catheter-days, n (95% CI) | 7.6 (4.4–10.8) | 4.2 (0.1–8.3) | 5.4 (1.1–9.8) | 14.3 (6.2–22.4) | 0.03 |
|  |  |  |  |  |  |
| CRBSI |  |  |  |  |  |
| Total |  |  |  |  |  |
| Number of catheters (%) | 12 (2.3%) | 4 (2.3%) | 3 (1.7%) | 5 (3.1%) | 0.69 |
| Incidence, per 1000 catheter-days, n (95% CI) | 4.1 (1.8–6.5) | 4.2 (0.1–8.3) | 2.7 (0–5.8) | 6.0 (0.7–11.2) | 0.60 |
| 1. Hazard ratio for each two antiseptic solution | |  |  |  |  |
| colonization | ≥72 h | |  |  |  |
|  | HR (95% CI) | P value |  |  |  |
| 0.5% CHG vs. 10% PVI | 0.28(0.09–0.90) | 0.03 |  |  |  |
| 1.0% CHG vs. 10% PVI | 0.37 (0.14–1.004) | 0.051 |  |  |  |
| 0.5% CHG vs. 1.0% CHG | 0.77 (0.22–2.73) | 0.68 |  |  |  |

CHG: chlorhexidine gluconate, PVI: povidone-iodine, HR: Hazard ratio IQR: interquartile range, CI: confidence interval, CRBSI: catheter-related blood stream infection

**Table S7 Catheter outcomes for central venous catheters excluding those inserted in the femoral vein (sensitivity analysis)**

|  | All  N = 762 | 0.5% CHG  N = 251 | 1.0% CHG  N = 268 | 10% PVI  N = 243 | P value |
| --- | --- | --- | --- | --- | --- |
| Colonization |  |  |  |  |  |
| Number of catheters (%) | 23 (3.0%) | 5 (2.0%) | 6 (2.2%) | 12 (4.9%) | 0.10 |
| Incidence, per 1000 catheter-days, n (95% CI) | 5.8 (3.4–8.2) | 3.8 (0.5–7.1) | 4.0 (0.8–7.2) | 10.3 (4.5–16.2) | 0.044 |
|  |  |  |  |  |  |
| CRBSI |  |  |  |  |  |
| Number of catheters (%) | 12 (1.6%) | 4 (1.6%) | 3 (1.1%) | 5 (2.1%) | 0.70 |
| Incidence, per 1000 catheter-days, n (95% CI) | 3.0 (1.3–4.7) | 3.0 (0.1–6.0) | 2.0 (0–4.3) | 4.3 (0.5–8.1) | 0.59 |
| 1. Hazard ratio for each two antiseptic solution |  |  |  |  |  |
|  | Colonization | | CRBSI | |  |
|  | HR (95% CI) | P value | HR (95% CI) | P value |  |
| 0.5% CHG vs. 10% PVI | 0.35 (0.11–0.95) | 0.04 | 0.69 (0.17–2.62) | 0.58 |  |
| 1.0% CHG vs. 10% PVI | 0.37 (0.13–0.97) | 0.04 | 0.48 (0.10–1.98) | 0.31 |  |
| 0.5% CHG vs. 1.0% CHG | 0.93 (0.27–3.09) | 0.90 | 1.43 (0.32–7.28) | 0.64 |  |

CHG: chlorhexidine gluconate, PVI: povidone-iodine, HR: hazard ratio, CI: confidence interval, CRBSI: catheter-related blood stream infection

**Table S8 Summary of patient characteristics and outcomes in patients with/without colonization and antibiotics before catheter insertion**

|  | Colonization (+) | | | Colonization (-) | | |
| --- | --- | --- | --- | --- | --- | --- |
|  | Total  N=24 | Antibiotics (+)  N=15 | Antibiotics (-)  N=9 | Total  N=772 | Antibiotics (+)  N=430 | Antibiotics (-)  N=342 |
| Age, mean (SD), years | 61.5 (16.9) | 57.6 (18.3) | 68.1 (12.5) | 66.4(16.3) | 67.6 (15.7) | 64.8 (16.8) |
| Gender, male (no., %) | 15 (62.5) | 8 (53.3) | 7 (77.8) | 489 (63.3) | 272 (63.3) | 217 (63.5) |
| APACHE 2, mean (SD) | 23.4 (6.5) | 22.7 (5.7) | 24.6 (7.9) | 20.9 (8.6) | 21.6 (8.5) | 20.1 (8.7) |
| SAPS 2, mean (SD) | 44.5 (14.3) | 42.3 (15.4) | 48.0 (12.3) | 49.6 (18.7) | 51.1 (18.4) | 47.7 (18.9) |
| SOFA, mean (SD) | 6.9 (3.9) | 7.1 (4.5) | 6.6 (2.9) | 7.2 (4.1) | 8.0 (4.1) | 6.2 (3.7) |
| Comorbidities (no., %) |  |  |  |  |  |  |
| Immunodeficiency | 2 (8.3) | 1 (6.7) | 1 (11.1) | 37 (4.8) | 28 (6.5) | 9 (2.6) |
| Corticosteroids administered | 2 (8.3) | 1 (6.7) | 1 (11.1) | 88 (11.4) | 59 (13.7) | 29 (8.5) |
| Trauma | 4 (16.7) | 2 (13.3) | 2 (22.2) | 60 (7.8) | 27 (6.3) | 33 (9.7) |
| Cancer | 0 (0) | 0 (0) | 0 (0) | 84 (10.9) | 63 (14.7) | 21 (6.1) |
| Diabetes | 5 (20.8) | 2 (13.3) | 3 (33.3) | 148 (19.2) | 94 (21.9) | 54 (15.8) |
| HIV | 0 (0) | 0 (0) | 0 (0) | 1 (0.1) | 1 (0.2) | 0 (0) |
| Cirrhosis | 1 (4.2) | 0(0) | 1 (11.1) | 39 (5.1) | 16 (3.7) | 23 (6.8) |
| Acute kidney injury | 6 (25.0) | 3 (20.0) | 3 (33.3) | 239 (31.0) | 152 (35.4) | 87 (25.4) |
| Chronic kidney disease | 4 (16.7) | 1 (6.7) | 3 (33.3) | 133 (17.2) | 90 (20.9) | 43 (12.6) |
| Admission category (no., %) |  |  |  |  |  |  |
| Medical | 15 (62.5) | 7 (46.7) | 8 (88.9) | 630 (81.6) | 348 (80.9) | 282 (82.4) |
| Scheduled surgery | 1 (4.2) | 1 (6.7) | 0 (0) | 22 (2.9) | 17 (4.0) | 5 (1.5) |
| Emergency surgery | 8 (33.3) | 7 (46.7) | 1 (11.1) | 120 (15.5) | 65 (15.1) | 55 (16.1) |
| Infections before catheter insertion (no., %) | 12 (50.0) | 10 (66.7) | 2 (22.2) | 436 (56.5) | 390 (90.7) | 46 (13.5) |
| Antiseptic solution used for cutaneous antisepsis |  |  |  |  |  |  |
| 0.5% CHG | 5 (20.8) | 5 (33.3) | 0 (0) | 256 (33.2) | 148 (34.4) | 108 (31.6) |
| 1.0% CHG | 6 (25.0) | 3 (20.0) | 3 (33.3) | 272 (35.2) | 152 (35.4) | 120 (35.1) |
| 10% PVI | 13 (54.2) | 7 (46.7) | 9 (66.7) | 244 (31.6) | 130 (30.2) | 114 (33.3) |
| Type of catheter (number, %) |  |  |  |  |  |  |
| Central venous catheter | 19 (79.2) | 14 (93.3) | 5 (55.6) | 266 (34.5) | 148 (34.4) | 118 (34.5) |
| Arterial catheter | 5 (20.8) | 1 (6.7) | 4 (44.4) | 506 (65.5) | 282 (65.6) | 224 (65.5) |
| Insertion site (number, %) |  |  |  |  |  |  |
| Central venous catheter |  |  |  |  |  |  |
| Internal jugular vein | 18 (94.7) | 13 (92.9) | 5 (100) | 247 (92.8) | 138 (93.2) | 109 (92.4) |
| Subclavian vein | 1 (5.3) | 1 (7.1) | 0 (0) | 10 (3.8) | 5 (3.4) | 5 (4.2) |
| Femoral vein | 0 (0) | 0 (0) | 0 (0) | 9 (3.4) | 5 (3.4) | 4 (3.4) |
| Arterial catheter |  |  |  |  |  |  |
| Radial artery | 4 (100) | 1 (100) | 3 (100) | 477 (96.0) | 259 (94.2) | 218 (98.2) |
| Femoral artery | 0 (0) | 0 (0) | 0 (0) | 15 (3.0) | 13 (4.7) | 2 (0.90 |
| Dorsalis artery | 0 (0) | 0 (0) | 0 (0) | 5 (1.0) | 3 (1.1) | 2 (0.9) |
| Duration of catheterization, median (IQR), days | 6.2 (3.8-11.8) | 6.7 (4.0-12.3) | 5.5 (3.5-7.6) | 3.8 (2.0-6.6) | 4.1 (2.1-7.0) | 3.4 (1.8-5.9) |
| Methods for catheter culture (number, %) |  |  |  |  |  |  |
| Maki methods | 18 (75.0) | 10 (66.7) | 8 (88.9) | 645 (83.6) | 369 (85.8) | 276 (80.7) |
| Sonication methods | 6 (25.0) | 5 (33.3) | 1 (11.1) | 127 (16.4) | 61 (14.2) | 65 (19.3) |

APACHE: acute physiology and chronic health evaluation, CHG: chlorhexidine gluconate, HIV: human immunodeficiency virus, IQR: interquartile range, PVI: povidone-iodine, SAPS: simplified acute physiology score, standard deviation, SD: standard deviation, SOFA: sequential organ failure assessment

**Table S9 Summary of patient characteristics and outcomes in patients with/without catheter-related blood stream infection and antibiotics before catheter insertion**

|  | CRBSI (+) | | | CRBSI (-) | | |
| --- | --- | --- | --- | --- | --- | --- |
|  | Total  N=13 | Antibiotics (+)  N=10 | Antibiotics (-)  N=3 | Total  N=783 | Antibiotics (+)  N=435 | Antibiotics (-)  N=348 |
| Age, mean (SD), years | 52.5 (19.0) | 49.3 (18.2) | 63.3 (21.6) | 66.5 (16.2) | 67.7 (15.6) | 64.9 (16.7) |
| Gender, male (no., %) | 4 (30.8) | 3 (30.0) | 1 (33.3) | 500 (63.9) | 277 (63.7) | 223 (64.1) |
| APACHE 2, mean (SD) | 24.2 (6.8) | 23.5 (6.3) | 26.3 (9.7) | 21.0 (8.5) | 21.6 (8.4) | 21.2 (8.7) |
| SAPS 2, mean (SD) | 57.8 (22.4) | 57.0 (21.7) | 60.3 (29.6) | 49.3 (18.5) | 50.6 (18.3) | 47.6 (18.7) |
| SOFA, mean (SD) | 12.9 (4.2) | 13.5 (4.4) | 11.0 (3.6) | 7.1 (4.0) | 7.9 (4.1) | 6.1 (3.7) |
| Comorbidities (no., %) |  |  |  |  |  |  |
| Immunodeficiency | 0 (0) | 0 (0) | 0 (0) | 39 (5.0) | 29 (6.7) | 10 (2.9) |
| Corticosteroids administered | 3 (23.1) | 2 (20.0) | 1 (33.3) | 87 (11.1) | 58 (13.3) | 29 (8.3) |
| Trauma | 1 (7.7) | 0 (0) | 1 (33.3) | 63 (8.1) | 29 (6.7) | 34 (9.8) |
| Cancer | 0 (0) | 0 (0) | 0 (0) | 84 (10.7) | 63 (14.5) | 21 (6.0) |
| Diabetes | 2 (15.4) | 1 (10.0) | 1 (33.3) | 151 (19.3) | 95 (21.8) | 56 (16.1) |
| HIV | 0 (0) | 0 (0) | 0 (0) | 1 (0.1) | 1 (0.2) | 0 (0) |
| Cirrhosis | 0 (0) | 0 (0) | 0 (0) | 40 (5.1) | 16 (3.7) | 24 (6.9) |
| Acute kidney injury | 5 (38.5) | 3 (30.0) | 2 (66.7) | 240 (30.7) | 152 (34.9) | 88 (25.3) |
| Chronic kidney disease | 1 (7.7) | 1 (10.0) | 0 (0) | 136 (17.4) | 90 (20.7) | 46 (13.2) |
| Admission category (no., %) |  |  |  |  |  |  |
| Medical | 6 (46.2) | 4 (40.0) | 2 (66.7) | 639 81.6) | 351 (80.7) | 288 (82.8) |
| Scheduled surgery | 0 (0) | 0 (0) | 0 (0) | 23 (2.9) | 18 (4.1) | 5 (1.4) |
| Emergency surgery | 7 (53.8) | 6 (60.0) | 1 (33.3) | 121 (15.5) | 66 (15.2) | 55 (15.8) |
| Infections before catheter insertion (no., %) | 11 (84.6) | 10 (100) | 1 (33.3) | 437 (55.8) | 390 (89.7) | 47 (13.5) |
| Antiseptic solution used for cutaneous antisepsis |  |  |  |  |  |  |
| 0.5% CHG | 4 (30.8) | 3 (30.0) | 1 (33.3) | 257 (32.8) | 150 (34.5) | 107 (30.7) |
| 1.0% CHG | 3 (23.1) | 2 (20.0) | 1 (33.3) | 275 (35.1) | 153 (35.2) | 122 (35.1) |
| 10% PVI | 6 (46.1) | 5 (50.0) | 1 (33.3) | 251 (32.1) | 132 (30.43 | 119 (34.2) |
| Type of catheter (number, %) |  |  |  |  |  |  |
| Central venous catheter | 8 (61.5) | 6 (60.0) | 2 (66.7) | 277 (35.4) | 156 (35.9) | 121 (34.8) |
| Arterial catheter | 5 (38.5) | 4 (40.0) | 1 (33.3) | 506 (64.6) | 279 (64.1) | 227 (65.2) |
| Insertion site (number, %) |  |  |  |  |  |  |
| Central venous catheter |  |  |  |  |  |  |
| Internal jugular vein | 7 (87.5) | 5 (83.3) | 2 (100) | 258 (93.1) | 146 (93.6) | 112 (92.6) |
| Subclavian vein | 1 (12.5) | 1 (16.7) | 0 (0) | 10 (3.6) | 5 (3.2) | 5 (4.1) |
| Femoral vein | 0 (0) | 0 (0) | 0 (0) | 9 (3.3) | 5 (3.2) | 4 (3.3) |
| Arterial catheter |  |  |  |  |  |  |
| Radial artery | 3 (60.0) | 3 (75.0) | 0 (0) | 478 (96.4) | 257 (94.5) | 221 (98.7) |
| Femoral artery | 1 (20.0) | 1 (25.0) | 0 (0) | 14 (2.8) | 12 (4.4) | 2 (0.9) |
| Dorsalis artery | 1 (20.0) | 0 (0) | 1 (100) | 4 (0.8) | 3 (1.1) | 1 (0.4) |
| Duration of catheterization, median (IQR), days | 8.0 (4.1-12.4) | 8.3 (4.2-14.5) | 8.0 (3.8-9.0) | 3.8 (2.0-6.6) | 4.1 (2.1-7.0) | 3.5 (1.9-5.9) |
| Methods for catheter culture (number, %) |  |  |  |  |  |  |
| Maki methods | 5 (38.5) | 4 (40.0) | 1 (33.3) | 658 (84.0) | 375 (86.2) | 283 (81.3) |

APACHE: acute physiology and chronic health evaluation, CHG: chlorhexidine gluconate, CRBSI: catheter-related blood stream infection, HIV: human immunodeficiency virus, IQR: interquartile range, PVI: povidone-iodine, SAPS: simplified acute physiology score, standard deviation, SD: standard deviation, SOFA: sequential organ failure assessment

**Table S10 Microorganisms isolated from catheter-tips in the three study groups**

|  | All  N = 796 | 0.5% CHG  N = 261 | 1.0% CHG  N = 278 | 10% PVI  N = 257 |
| --- | --- | --- | --- | --- |
| Total | 31 (3.9%) | 6 (2.3%) | 8 (2.9%) | 17 (6.6%) |
| Coagulase-negative Staphylococci | 19 (61.3%) | 3 (50.0%) | 7 (87.5%) | 9 (52.9%) |
| *Staphylococcus* *aureus* | 2 (6.5%) | 0 | 0 | 2 (11.8%) |
| *Pseudomonas aeruginosa* | 2 (6.5%) | 0 | 1 (12.5%) | 1 (5.9%) |
| *Enterococcus* species | 1 (3.2%) | 0 | 0 | 1 (5.9%) |
| *Enterobacter* species | 2 (6.5%) | 1 (16.7%) | 0 | 1 (5.9%) |
| *Candida* species | 3 (9.7%) | 2 (33.3%) | 0 | 1 (5.9%) |
| *Bacillus* species | 1 (3.2%) | 0 | 0 | 1 (5.9%) |
| *Serratia marcescens* | 1 (3.2%) | 0 | 0 | 1 (5.9%) |

Data were no. (%).

CHG: chlorhexidine gluconate, PVI: povidone-iodine

**Table S11 Microorganisms isolated in catheter-related bloodstream infections in the three study groups**

|  | All  N = 796 | 0.5% CHG  N = 261 | 1.0% CHG  N = 278 | 10% PVI  N = 257 |
| --- | --- | --- | --- | --- |
| Total | 14 (1.8%) | 4 (1.5%) | 3 (1.1%) | 7 (2.7%) |
| Coagulase-negative Staphylococci | 3 (21.4%) | 0 | 1 (33.3%) | 1 (14.3%) |
| *Staphylococcus aureus* | 2 (14.3%) | 1 (25%) | 0 | 2 (28.6%) |
| *Enterococcus* species | 1 (7.1%) | 0 | 0 | 1 (14.3%) |
| *Enterobacter* species | 3 (21.4%) | 2 (50%) | 0 | 1 (14.3%) |
| *Candida* species | 4 (28.6%) | 1 (25%) | 2 (66.7%) | 1 (14.3%) |
| *Streptococcus agalactiae* | 1 (7.1%) | 0 | 0 | 1 (14.3%) |

Data are shown as number (%).

CHG: chlorhexidine gluconate, PVI: povidone-iodine,

Table S**12 Adverse events**

| Characteristics | All  N = 796 | 0.5% CHG  N = 261 | 1.0% CHG  N = 278 | 10% PVI  N = 257 | P value |
| --- | --- | --- | --- | --- | --- |
| Adverse reactions due to antiseptic solution | 0 (0%) | 0 (0%) | 0 (0%) | 0 (0%) | - |
| Redness | 0 (0%) | 0 (0%) | 0 (0%) | 0 (0%) | - |
| Hives | 0 (0%) | 0 (0%) | 0 (0%) | 0 (0%) | - |
| Anaphylaxis | 0 (0%) | 0 (0%) | 0 (0%) | 0 (0%) | - |
| Catheter insertion site findings |  |  |  |  |  |
| Redness | 6 (0.8%) | 2 (0.8%) | 0 (0%) | 4 (1.6%) | 0.12 |
| Heat sensation | 2 (0.3%) | 0 (0%) | 0 (0%) | 2 (0.8%) | 0.12 |
| Swelling | 1 (0.1%) | 0 (0%) | 0 (0%) | 1 (0.4%) | 0.35 |
| Pain | 3 (0.4%) | 1 (0.4%) | 0 (0%) | 2 (0.8%) | 0.34 |
| Purulent exudate | 1 (0.1%) | 0 (0%) | 1 (0.4%) | 0 (0%) | 0.39 |

Data are shown as number (%).

CHG: chlorhexidine gluconate, PVI: povidone-iodine

**Table S13 Patient outcomes**

|  | All  N = 796 | 0.5% CHG  N = 261 | 1.0% CHG  N = 278 | 10% PVI  N = 257 | P value |
| --- | --- | --- | --- | --- | --- |
| ICU length of stay, median (IQR), days | 7.1 (3.3–17.3) | 7.0 (3.5–16.2) | 7.1 (3.5–17.2) | 7.1 (3.2–17.9) | 0.99 |
| Hospital length of stay, median (IQR), days | 34.0 (15.3–70.0) | 34.8 (15.7–65.2) | 33.9 (16.8–68.0) | 32.5 (13.9–71.7) | 0.87 |
| Intensive Care Unit mortality (number, %) | 158 (19.9%) | 51 (19.5%) | 47 (16.9%) | 60 (23.4%) | 0.17 |
| Hospital mortality (number, %) | 237 (29.8%) | 75 (28.7%) | 81 (29.1%) | 81 (31.5%) | 0.76 |

CHG: chlorhexidine gluconate, PVI: povidone-iodine, IQR: interquartile range, ICU: Intensive Care Unit

**Table S14 Proportion of missing data for each variable in the three study groups**

1. **Full Analysis Set**

|  | All  N = 796 | 0.5% CHG  N = 261 | 1.0% CHG  N = 278 | 10% PVI  N = 257 |
| --- | --- | --- | --- | --- |
| Age | 0 | 0 | 0 | 0 |
| Gender | 0 | 0 | 0 | 0 |
| APACHE 2 | 1 (0.1%) | 0 | 0 | 1 (0.4%) |
| SAPS 2 | 1 (0.1%) | 0 | 0 | 1 (0.4%) |
| SOFA | 1 (0.1%) | 0 | 0 | 1 (0.4%) |
| Comorbidities |  |  |  |  |
| Immunodeficiency | 1 (0.1%) | 0 | 1 (0.4%) | 0 |
| Steroid | 0 | 0 | 0 | 0 |
| Trauma | 0 | 0 | 0 | 0 |
| Cancer | 0 | 0 | 0 | 0 |
| Diabetes | 0 | 0 | 0 | 0 |
| HIV | 26 (3.3%) | 7 (2.7%) | 10 (3.6%) | 9 (3.5%) |
| Cirrhosis | 1 (0.1%) | 1 (0.4%) | 0 | 0 |
| Acute kidney injury | 0 | 0 | 0 | 0 |
| Chronic kidney disease | 0 | 0 | 0 | 0 |
| Admission category | 0 | 0 | 0 | 0 |
| Infections before catheter insertion | 0 | 0 | 0 | 0 |
| Origin of infection | 1 (0.1%) | 1 (0.4%) | 0 | 0 |
| Antibiotics before catheter insertion | 0 | 0 | 0 | 0 |
| Type of catheter | 0 | 0 | 0 | 0 |
| Insertion site | 10 (1.3%) | 3 (1.2%) | 3 (1.1%) | 4 (1.6%) |
| Central venous catheter | 0 | 0 | 0 | 0 |
| Arterial catheter | 10 (100%) | 3 (100%) | 3 (100%) | 4 (100%) |
| Duration of catheterization | 1 (0.1%) | 1 (0.4%) | 0 (0%) | 0 (0%) |
| Methods for catheter culture | 0 | 0 | 0 | 0 |
| Dressing | 0 | 0 | 0 | 0 |
| Adverse effects due to antiseptic solution |  |  |  |  |
| Redness | 0 | 0 | 0 | 0 |
| Hives | 0 | 0 | 0 | 0 |
| Anaphylaxis | 0 | 0 | 0 | 0 |
| Catheter insertion site findings |  |  |  |  |
| Redness | 0 | 0 | 0 | 0 |
| Heat sensation | 0 | 0 | 0 | 0 |
| Swelling | 0 | 0 | 0 | 0 |
| Pain | 0 | 0 | 0 | 0 |
| Purulent exudate | 0 | 0 | 0 | 0 |
| Intensive Care Unit length of stay | 0 | 0 | 0 | 0 |
| Hospital length of stay | 0 | 0 | 0 | 0 |
| Intensive Care Unit mortality | 0 | 0 | 0 | 0 |
| Hospital mortality | 0 | 0 | 0 | 0 |
| Catheter colonization | 0 | 0 | 0 | 0 |
| CRBSI | 0 | 0 | 0 | 0 |

Data are shown as: number (%).

CHG: chlorhexidine gluconate, PVI: povidone-iodine, HR: hazard ratio, CRBSI: catheter-related bloodstream infection, APACHE: acute physiology and chronic health evaluation, SAPS: simplified acute physiology score, SOFA: sequential organ failure assessment

1. Intention to treat

|  | All  N = 998 | 0.5% CHG  N = 329 | 1.0% CHG  N = 339 | 10% PVI  N = 330 |
| --- | --- | --- | --- | --- |
| Age | 0 | 0 | 0 | 0 |
| Gender | 0 | 0 | 0 | 0 |
| APACHE 2 | 1 (0.1%) | 0 | 0 | 1 (0.3%) |
| SAPS 2 | 1 (0.1%) | 0 | 0 | 1 (0.3%) |
| SOFA | 1 (0.1%) | 0 | 0 | 1 (0.3%) |
| Comorbidities |  |  |  |  |
| Immunodeficiency | 1 (0.1%) | 0 | 1 (0.3%) | 0 |
| Corticosteroids administered | 0 | 0 | 0 | 0 |
| Trauma | 0 | 0 | 0 | 0 |
| Cancer | 0 | 0 | 0 | 0 |
| Diabetes | 0 | 0 | 0 | 0 |
| HIV | 27 (2.7%) | 8 (2.4%) | 10 (3.0%) | 9 (2.7%) |
| Cirrhosis | 1 (0.1%) | 1 (0.3%) | 0 | 0 |
| Acute kidney injury | 0 | 0 | 0 | 0 |
| Chronic kidney disease | 0 | 0 | 0 | 0 |
| Admission category | 0 | 0 | 0 | 0 |
| Infections before catheter insertion | 0 | 0 | 0 | 0 |
| Origin of infection | 1 (0.1%) | 1 (0.3%) | 0 | 0 |
| Antibiotics before catheter insertion | 0 | 0 | 0 | 0 |
| Type of catheter | 0 | 0 | 0 | 0 |
| Insertion site | 12 (1.2%) | 3 (0.9%) | 3 (0.9%) | 6 (1.8%) |
| Central venous catheter | 0 | 0 | 0 | 0 |
| Arterial catheter | 12 (100%) | 3 (100%) | 3 (100%) | 6 (100%) |
| Duration of catheterization | 55 (5.5%) | 22 (6.7%) | 15 (4.4%) | 18 (5.5%) |
| Methods for catheter culture | 0 | 0 | 0 | 0 |
| Dressing | 1 (0.1%) | 0 | 0 | 1 (0.3%) |
| Adverse effects due to antiseptic solution |  |  |  |  |
| Redness | 1 (0.1%) | 0 | 0 | 1 (0.3%) |
| Hives | 1 (0.1%) | 0 | 0 | 1 (0.3%) |
| Anaphylaxis | 1 (0.1%) | 0 | 0 | 1 (0.3%) |
| Catheter insertion site findings |  |  |  |  |
| Redness | 1 (0.1%) | 0 | 0 | 1 (0.3%) |
| Heat sensation | 1 (0.1%) | 0 | 0 | 1 (0.3%) |
| Swelling | 1 (0.1%) | 0 | 0 | 1 (0.3%) |
| Pain | 1 (0.1%) | 0 | 0 | 1 (0.3%) |
| Purulent exudate | 1 (0.1%) | 0 | 0 | 1 (0.3%) |
| Intensive Care Unit length of stay | 0 | 0 | 0 | 0 |
| Hospital length of stay | 0 | 0 | 0 | 0 |
| Intensive Care Unit mortality | 0 | 0 | 0 | 0 |
| Hospital mortality | 0 | 0 | 0 | 0 |
| Catheter colonization |  |  |  |  |
| Total | 202 (20.2%) | 68 (20.7%) | 61 (17.7%) | 73 (21.1%) |
| Central venous catheter | 56 (27.7%) | 20 (29.4%) | 20 (32.8%) | 16 (21.9%) |
| Arterial catheter | 146 (72.3%) | 48 (70.6%) | 41 (67.2%) | 57 (78.1%) |
| CRBSI |  |  |  |  |
| Total | 2 (0.2%) | 1 (0.3%) | 0 | 1 (0.3%) |
| Central venous catheter | 0 | 0 | 0 | 0 |
| Arterial catheter | 2 (100%) | 1 (100%) | 0 | 1 (100%) |

Data are shown as: number (%).

CHG: chlorhexidine gluconate, PVI: povidone-iodine, HR: hazard ratio, CRBSI: catheter-related bloodstream infection, APACHE: acute physiology and chronic health evaluation, SAPS: simplified acute physiology score, SOFA: sequential organ failure assessment, HIV: human immunodeficiency virus

**Table S15 The specific names of all ethical bodies that approved this study in various participating sites**

|  | Participating site | Committee Name | Approval number |
| --- | --- | --- | --- |
| 1 | Japanese Red Cross Musashino Hospital | Clinical Research Judging Committee | 521 |
| 2 | Saitama Medical Center, Jichi Medical University | Bioethics Committee for Clinical Research | CL12-47 |
| 3 | Yokohama City Minato Red Cross Hospital | Medical Ethics Committee | 2012-11 |
| 4 | Kurashiki Central Hospital | The Medical Ethics Committee | 1177 |
| 5 | Jikei University School of Medicine | The Ethics committee of The Jikei University School of Medicine for Biomedical Research | 24-090 6856 |
| 6 | Toyonaka Municipal Hospital | Ethics Committee of Toyonaka Municipal Hospital | Not assigned |
| 7 | Sendai City Hospital | Institutional review board by Sendai city hospital | 2012,12,11 Senbyoso No479 |
| 8 | Kobe City Medical Center General Hospital | Kobe City Medical Center General Hospital Institutional Review Board | 1212-05 |
| 9 | National Hospital Organization Kyoto Medical Center | Kyoto medical center ethical review board | 12-61 |
| 10 | Kyushu University Hospital | Center of Clinical and Translational Research, Kyushu University | 24106 |
| 11 | Nippon Medical School Chiba Hokusoh Hospital | Ethics committee | No.330 |
| 12 | Okayama Medical Center | Clinical Research Review Board Committee | H24-Clinical Research-54 |
| 13 | Tokyo Bay Urayasu Ichikawa Medical Center | Institutional Review Board of Tokyo Bay Urayasu Ichikawa Medical Center | 12 |
| 14 | Tohoku Medical and Pharmaceutical University Hospital | Clinical Research Review Board Committee | Not assigned |
| 15 | Saiseikai Kumamoto Hospital | medical ethics committee | 298 |
| 16 | Takarazuka City Hospital | Hospital Ethics Committee | 122 |
