# Supplementary figures and images for: Comparison of the efficacy of three topical antiseptic solutions for the prevention of catheter colonization: a multicenter randomized controlled study
Source: Crit Care. 2017 Dec 21;21:320. doi: 10.1186/s13054-017-1890-z (PMC5740719; doi:10.1186/s13054-017-1890-z)

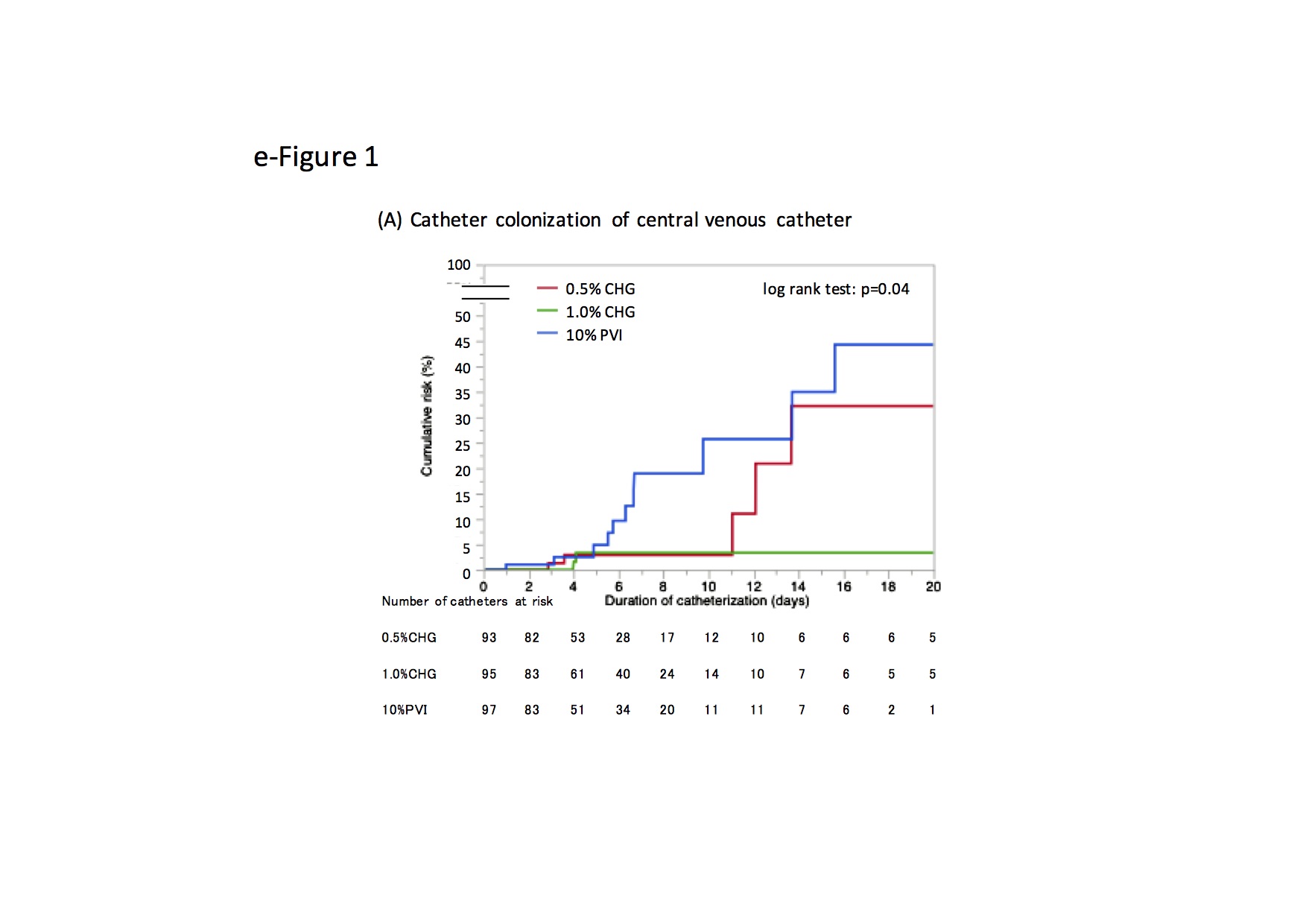

Supplement: Supplementary file 2 — (A) Colonization of central venous catheters. Cumulative catheter colonization and catheter-related bloodstream infection risk in each subgroup (Kaplan–Meier curves). CHG chlorhexidine gluconate, PVI povidone-iodine. (JPG 108 kb) [file 13054_2017_1890_MOESM2_ESM.jpg]

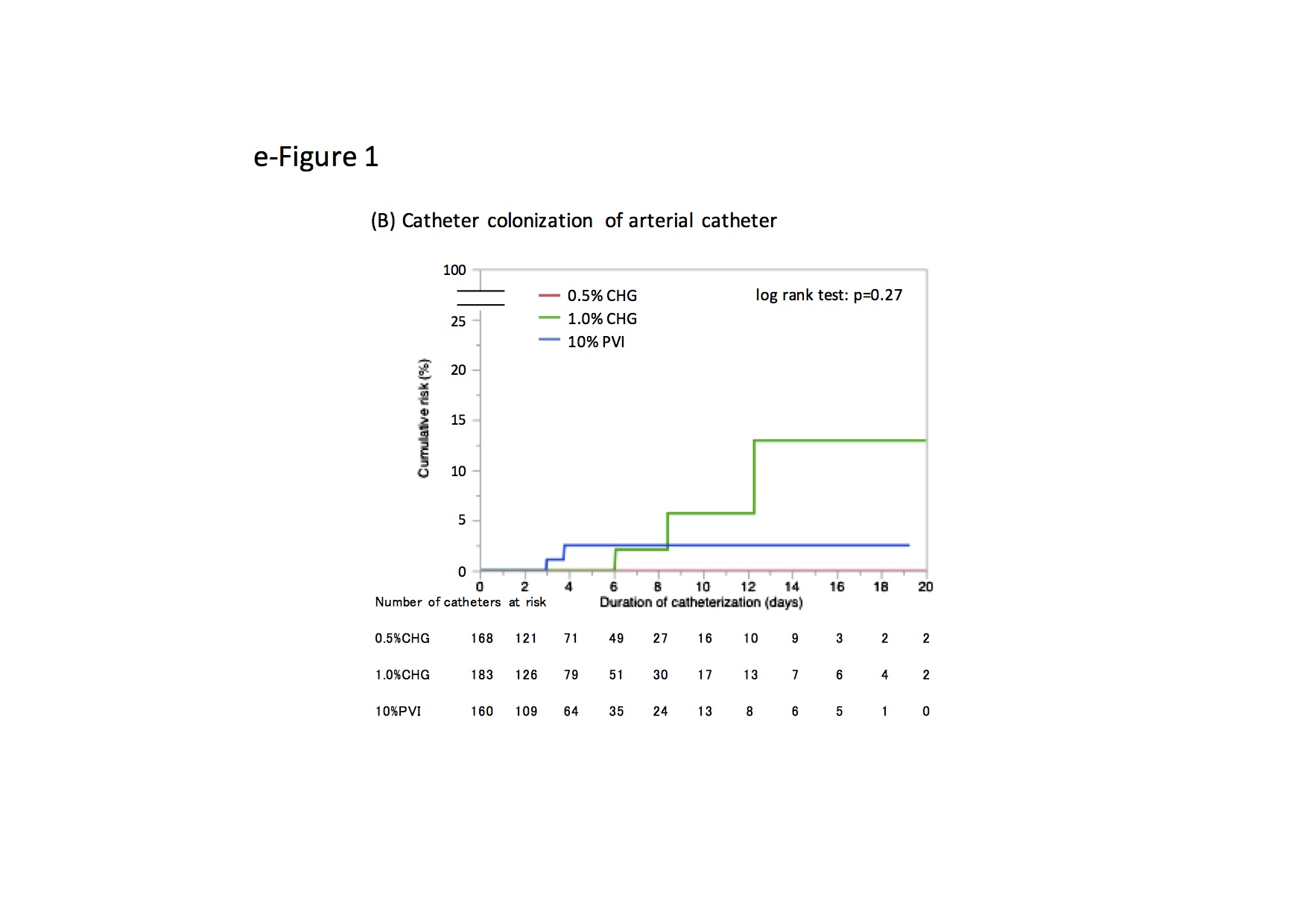

Supplement: Supplementary file 3 — (B) Colonization of arterial catheters. Cumulative catheter colonization and catheter-related bloodstream infection risk in each subgroup (Kaplan–Meier curves). CHG chlorhexidine gluconate, PVI povidone-iodine. (JPG 102 kb) [file 13054_2017_1890_MOESM3_ESM.jpg]

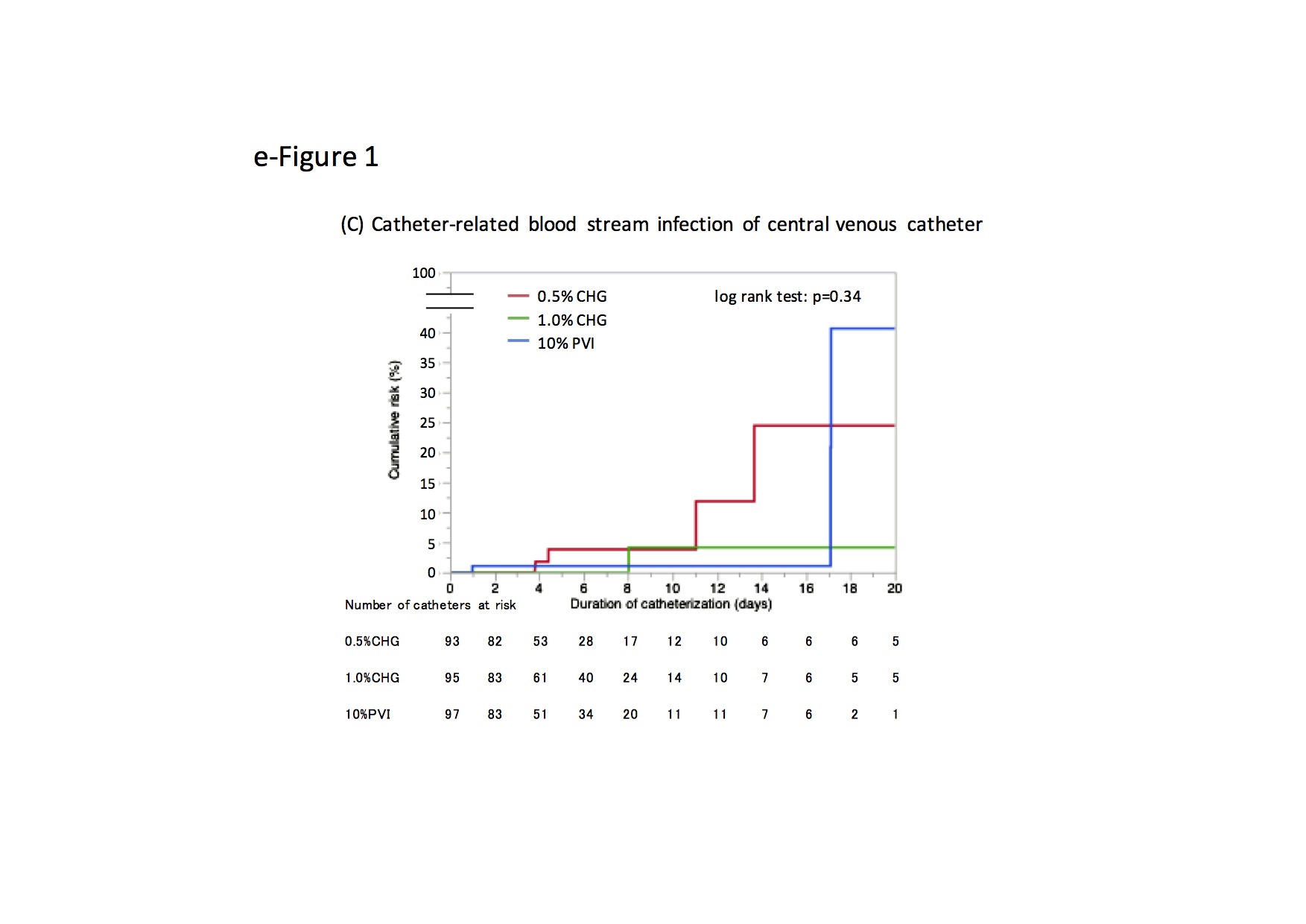

Supplement: Supplementary file 4 — (C) Catheter-related bloodstream infection of central venous catheters. Cumulative catheter colonization and catheter-related bloodstream infection risk in each subgroup (Kaplan–Meier curves). CHG chlorhexidine gluconate, PVI povidone-iodine. (JPG 116 kb) [file 13054_2017_1890_MOESM4_ESM.jpg]

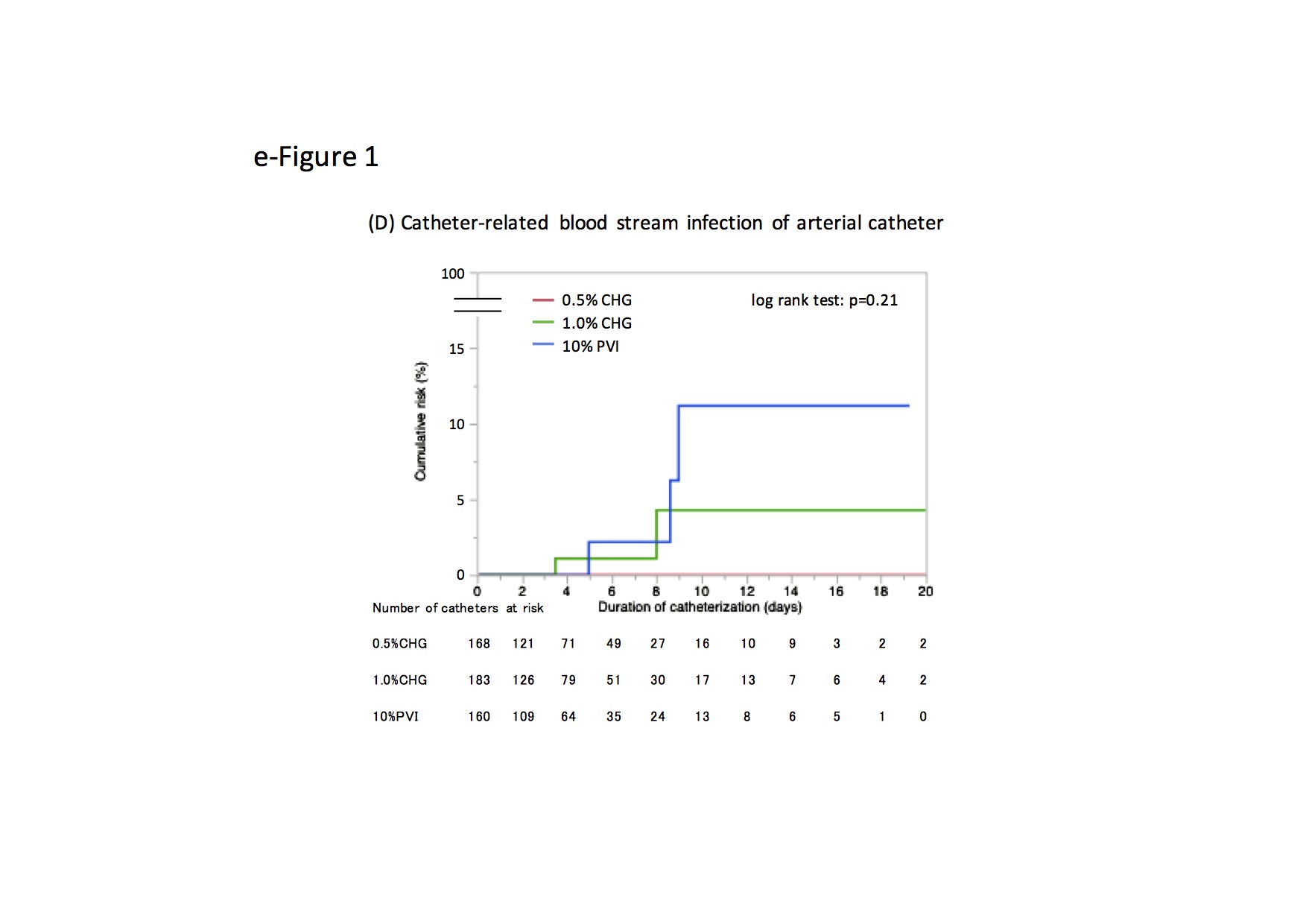

Supplement: Supplementary file 5 — (D) Catheter-related bloodstream infection of arterial catheters. Cumulative catheter colonization and catheter-related bloodstream infection risk in each subgroup (Kaplan–Meier curves). CHG chlorhexidine gluconate, PVI povidone-iodine (JPG 108 kb) [file 13054_2017_1890_MOESM5_ESM.jpg]

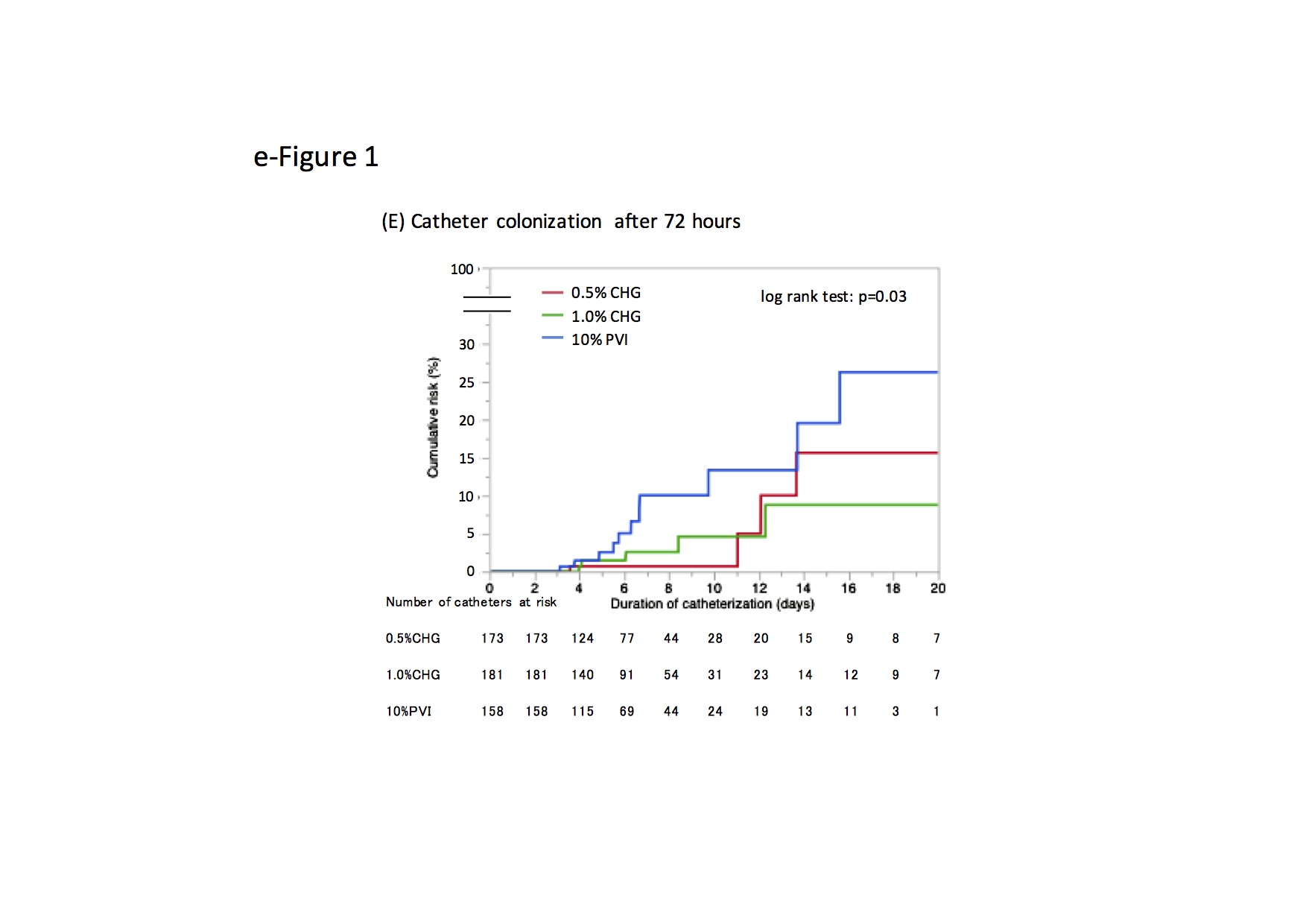

Supplement: Supplementary file 6 — (E) Catheter colonization after 72 h. Cumulative catheter colonization and catheter-related bloodstream infection risk in each subgroup (Kaplan–Meier curves). CHG chlorhexidine gluconate, PVI povidone-iodine. (JPG 107 kb) [file 13054_2017_1890_MOESM6_ESM.jpg]

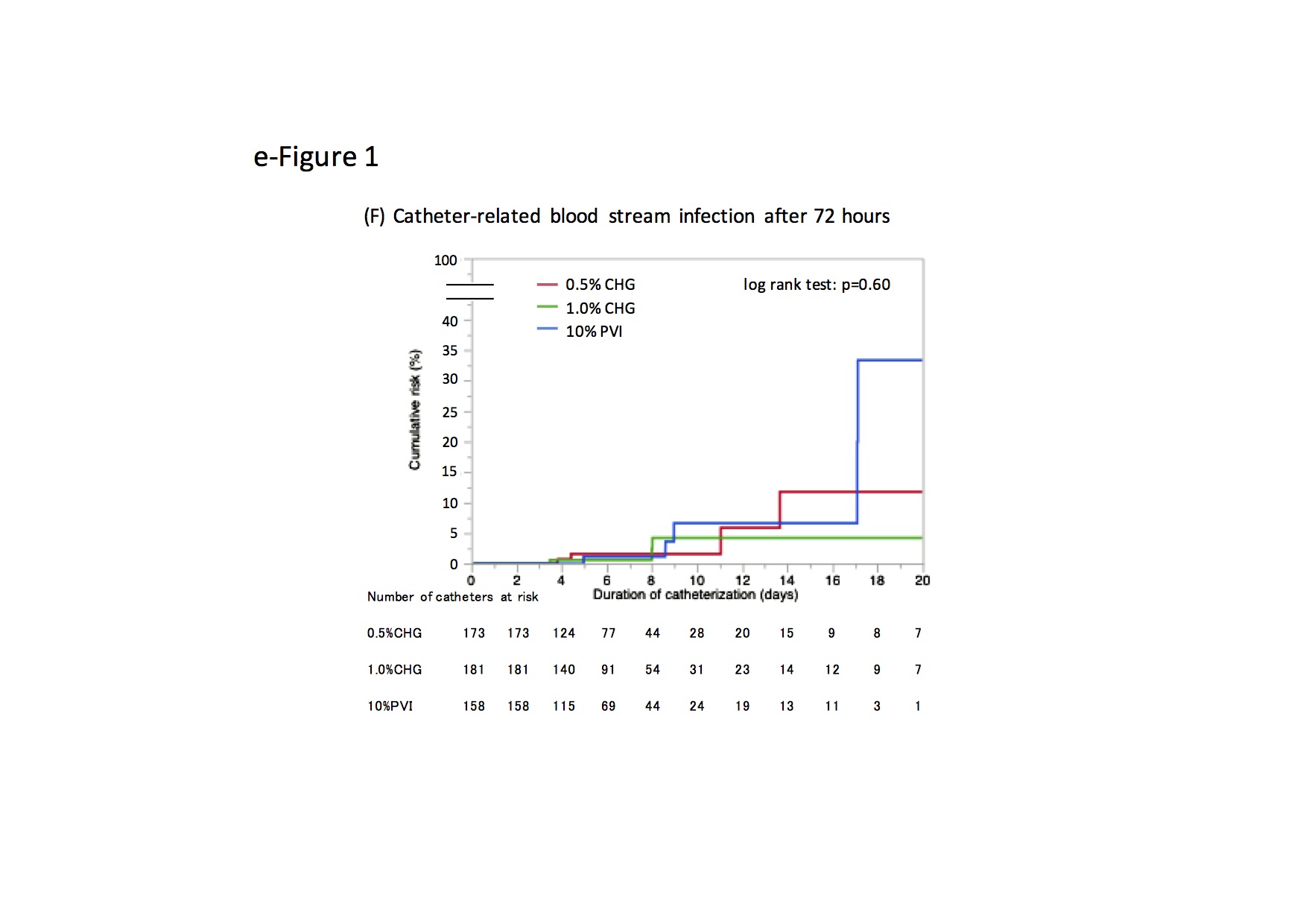

Supplement: Supplementary file 7 — (F) Catheter-related bloodstream infections after 72 h. Cumulative catheter colonization and catheter-related bloodstream infection risk in each subgroup (Kaplan–Meier curves). CHG chlorhexidine gluconate, PVI povidone-iodine. (JPG 115 kb) [file 13054_2017_1890_MOESM7_ESM.jpg]
